# The effectiveness of prehabilitation on post-operative recovery from lumbar spinal stenosis surgery – A systematic review and intervention component analysis

**DOI:** 10.1177/02692155261418206

**Published:** 2026-03-14

**Authors:** Peter Heine, Rebecca Hunter, Andrew Booth, Sarah E Lamb, Esther Williamson, Opinder Sahota, Bethan E Phillips, Paul Hendrick, Lianne Wood

**Affiliations:** 1 Department of Public Health & Sports Sciences, 3286 University of Exeter, Exeter, UK; 2 Sheffield Centre for Health and Related Research (SCHARR), School of Medicine and Population Health, 7315 University of Sheffield, Sheffield, UK; 3 Nuffield Department of Orthopaedics, Rheumatology and Musculoskeletal Sciences, 6396 University of Oxford, Oxford, UK; 4 Spinal Services, 9820 Nottingham University Hospitals NHS Trust, Nottingham, UK; 5 School of Medicine, 6123 University of Nottingham, Nottingham, UK

**Keywords:** Prehabilitation, lumbar spinal stenosis, neurogenic claudication, spinal surgery

## Abstract

**Objective:**

To evaluate prehabilitation interventions evaluated in randomised controlled trials for people undergoing lumbar spinal stenosis surgery and determine which individual intervention components are associated with successful interventions.

**Data sources:**

English language papers from 2011 until December 2025 from PubMed, Cumulative Index of Nursing and Allied Health, Scopus and Web of Science.

**Review methods:**

We searched for randomised controlled trials comparing prehabilitation to other non-active interventions for people undergoing surgery for lumbar spinal stenosis. Two authors independently screened, selected and performed quality assessments of the studies. Relevant study details were extracted, tabulated and synthesised using intervention component analysis.

**Results:**

Nine papers describing five randomised control trials involving 466 participants and 28 outcomes measured pre- and/or post-surgery were found. Overall, the interventions consisted of 47 individual components. Fifteen of these components (including psychological/behavioural approaches, and cardiovascular, strength and trunk/core exercise) featured in successful interventions that resulted in greater improvement in 16 outcomes (including back pain, disability, walking and hospital stay) in prehabilitation compared to usual care participants. The results of further evaluation of individual outcomes at each time point was inconsistent and unreliable.

**PROSPERO registration number:**

CRD42025645253, https://www.crd.york.ac.uk/PROSPERO/view/CRD42025645253

**Conclusion:**

Only five small trials of low-to-moderate quality report common outcomes at similar timepoints. There is high uncertainty regarding the importance of individual intervention components in successful prehabilitation interventions for people undergoing lumbar spinal stenosis surgery.

## Introduction

Neurogenic claudication resulting from lumbar spinal stenosis is a leading cause of spinal surgery in older adults.^1^ Symptoms may include leg pain, muscle weakness and impaired sensation resulting in pain and substantial disability, especially with standing and walking.^2^ About 11% of community dwelling older adults experience symptoms attributed to lumbar spinal stenosis.^3^ For most people, symptoms can be managed conservatively but, for a small proportion, spinal surgery may be offered to decompress the spinal canal to take the pressure off the nerve roots.^4^

In the UK, people may wait on average three to 12 months for elective spinal surgery.^5^ Prolonged waits for spinal surgery have been shown to be an independent predictor of poor surgical outcomes^6^ and can also impact people's mental and physical health while they wait. A review of patients in the British Spinal Registry undergoing surgery for lumbar spinal stenosis suggests that patients in the UK tend to be older, present with more comorbidities and report higher levels of pre-operative pain and disability compared to those in other countries.^7^ These factors are also associated with poorer post-surgical outcomes, longer hospital stays and greater likelihood of surgical complications.^8–11^

Prehabilitation is any intervention prior to surgery designed to maximise pre-surgical physical and/or mental health, surgical preparedness and subsequent post-surgical outcomes.^12^ Across different clinical conditions, prehabilitation compared to usual care appears to demonstrate promise in reducing surgical complications, length of stay in hospital and improving health-related quality of life and physical recovery.^13^ Only a few studies have explored the effects of prehabilitation in lumbar spinal stenosis, with recent scoping reviews supporting the general benefit of supervised prehabilitation interventions in comparison to usual care when prehabilitation is individualised, multimodal and interdisciplinary.^14^ In contrast, a systematic review and meta-analysis of prehabilitation for any elective lumbar spinal procedure reported considerable uncertainty, low quality and heterogeneity across the eight included trials (739 participants) which prevented clinical recommendations.^15^ Other systematic reviews conducted in this area report low-to-moderate evidence for prehabilitation prior to lumbar spinal surgery for a small number of outcomes.^16^ Importantly, the interventions described by the previous reviews of prehabilitation for spinal surgery varied in terms of content, structure, duration and delivery methods.

Our team has previously undertaken a realist review which generated programme theories for how prehabilitation may facilitate patient engagement in people undergoing surgery for lumbar spinal stenosis.^17^ However, it is necessary to understand which intervention components are critical to success within the design of an optimal prehabilitation intervention. This review aims to identify key components of prehabilitation programmes associated with improved outcomes in people undergoing surgery for lumbar spinal stenosis.

## Methods

We searched for eligible papers published in English from January 2011 to 1 December 2025 from PubMed, Cumulative Index of Nursing and Allied Health, Scopus and Web of Science (Supplementary information: A). The searches included free text terms ‘prehabilitation’, ‘preoperative’ and ‘peri-operative rehabilitation’ as well as relevant subject headings available in each database (e.g., Medical Subject Headings). We also performed backward and forward citation tracking by examining reference lists and searching citations of included papers for additional eligible studies.

Criteria for study inclusion are listed in Table 1 along with primary and secondary outcomes. After duplicates were removed, title and abstract screening was conducted by two authors (PH, LW) followed by full text review of suitable studies. Any discrepancies were resolved by discussion. The Cochrane Risk of Bias Tool (version 2) was used to assess risk of bias for each of five domains along with an overall judgement for each study. Studies were rated as either ‘low’, ‘some concerns’, or ‘high’.^18^ The Template for Intervention Description and Replication checklist^19^ was used to evaluate the quality of reporting for each study, although no explicit scoring mechanism was used due to the lack of evidence as to its validity.^20^

**Table 1.** Study inclusion eligibility criteria.

Category	Inclusion criteria	Exclusion criteria
Population	People ≥50 years who had undergone surgery for LSS with associated neurogenic claudication due to degenerative causes	Mean age <50 yrs of age; studies that excluded people with predominantly leg symptoms
Intervention(s)	Any pre-operative intervention in any setting whose aim was to optimise perioperative and/or post-surgical outcomes	Interventions consisting of single session of education or advice
Comparator	Usual care (no treatment or basic information/education only)	Other prehabilitation interventions that do not equate to usual care as defined here
Outcome(s)	*Primary:* symptom severity (leg and/or back pain), disability and walking outcomes. *Other:* quality of life, global improvement, self-efficacy, fear-avoidance, satisfaction, surgical complications, adverse events, hospital length of stay, failure/re-operation rate, return to work/work status/absenteeism, resource use, medication use, drop-out rate, adherence.	Trials which did not report at least one of listed outcomes
Studies	Randomised controlled trials published in English after 2010	Non-randomised studies; non-English publications; before 2011

Study characteristics, participant details, outcome measures, results and intervention details were extracted from each study and tabulated. An Intervention Component Analysis approach was used to identify the association of intervention components with outcome results, where possible. The Intervention Component Analysis approach involves a two-step process: (1) identify the individual intervention components from each study intervention; (2) identify which study interventions, and their constituent components, resulted in successful or unsuccessful treatment effects.^21^ We used a modified version of Intervention Component Analysis similar to a previous review of exercise interventions in lumbar spinal stenosis.^22^ Further details of the methods used and the mapping of the intervention onto study outcomes are described in Supplementary information: B.

The protocol for this review was registered with PROSPERO (CRD42025645253)^23^ and reported according to the Preferred Reporting Items for Systematic Reviews and Meta-Analyses guidelines.^24^

## Results

**Figure 1.**
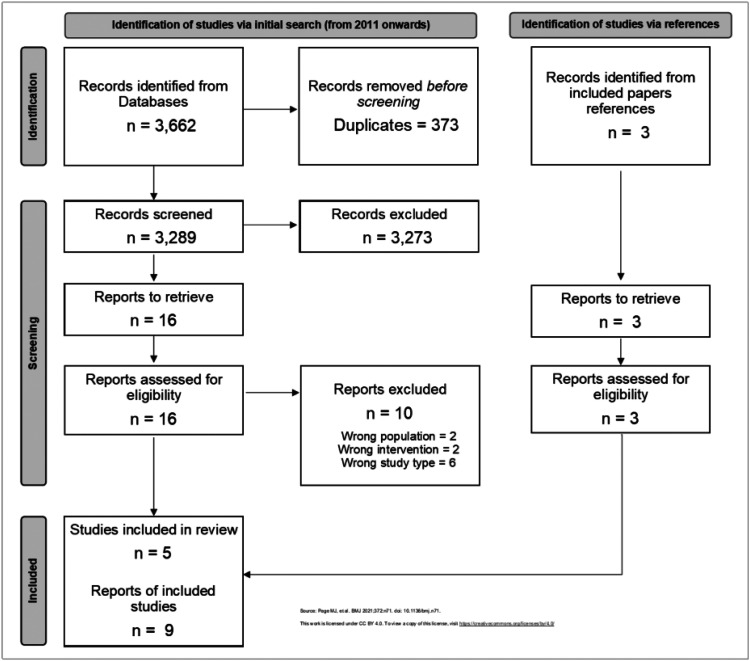
Screening results.

After abstract and title screening, full text review and examination of references and citations, nine publications providing unique data regarding five trials were included from the 3662 papers identified in the initial searches (Figure 1).

Only two trials specifically evaluated prehabilitation in lumbar spinal stenosis.^25,26^ The remaining three trials involved participants undergoing surgery for various degenerative spinal conditions including lumbar spinal stenosis.^27–29^

The five included trials randomised 466 participants (52% female) into either prehabilitation (*n* = 247) or various types of usual pre-surgical care (*n* = 219). The average age ranged from 48 to 71 years although participants as young as 23 years were included. Details regarding setting, sample size, intervention duration and follow-up period are described in Table 2.

**Table 2.** Summary of included trials.

Study	Participants	Interventions	Component	Further details/justification reported
Nielsen 2008, [30] 2010*[27] Denmark	73 randomised (35 I, 38 C), 60 FU/analysed (28 I, 32 C) [3 operated elsewhere (1 I, 2 C), 6 cancelled (2 I, 4 C), 4 withdrew consent (4 I, 0 C)] Female: 21/35 (61%) I, 22/38 (59%) C median age (range): 48 yrs (31–80) I, 52 yrs (23–88) C elective lumbar spinal surgery for degenerative disease with low back pain and radiating pain Primary spondylodesis instrumental 2/28 (7%) I, 2/32 (6%) C, Non-instrumental 23/28 (82%) I, 26/32 (81%) C, Secondary spondylodesis (non-instrumental) 2/28 (7%) I, 3/32 (9%) C, Implementation of discus prosthesis 1/28 (4%) I, 1/32 (3%) C	**Intervention:** Home exercise programme including abdominal/back strength & CV training with 1× monitoring phone call (2 wks pre-surgery), 6–8 wks, daily; 30 min smoking cessation programme (with free nicotine replacement therapy & weekly follow-up visits)^b^; 6 wks alcohol abstinence programme (with free medication & weekly follow-up visits)^c^, 4wks Optimised pain relief 200 ml protein drink evening prior to surgery preoperative information about surgery, anaesthetic procedure, medication, postoperative rehabilitation & physical restrictions after surgery. *Post-surgery:* inpatient rehab TWICE daily for 30 min 4 × 150mLprotein-rich drinks/day during inpatient stay optimised inpatient pain relief follow-up visits smoking cessation and alcohol abstinence programme at 3 & 6 mths. **Usual care:** Preoperative information about surgery, anaesthetic procedure, medication, postoperative rehabilitation & physical restrictions after surgery. *Post-surgery:* Inpatient rehab ONCE daily for 30 min routine pain relief	Exercise (CV, strength, trunk) (unsupervised) (individual) (HEP) Psychological/behavioural (supervised) (individual); Analgesia Nutrition/diet Usual care (education) Inpatient rehabilitation Nutrition/diet Analgesia Psychological/behavioural (supervised) (group) Usual care (education) Usual care (rehabilitation) Usual care (analgesia)	A
Rolving 2015* [33] 2016 [28] 2016b [34] Denmark	96 randomised (63 I, 33 C) 90 baseline (59 I, 31 C) 87 3&6 mth FU (58 I, 29 C) 83 12 mth FU (55 I, 28 C) 6 were excluded (4 I, 2C) because of changed/cancelled surgery female 36/59 (61%) I, 15/31 (48%) C age mean (SD) 51.4 yrs (9.2) I, 47.7 yrs (8.9) spondylolisthesis grades 1–2 16/59 (27%) I, 7/31 (23%) C degenerative disc disease 43/59 (73%,) I 24/31 (77%) C fusion of a maximum of 3 adjacent vertebrae posterolateral fusion 41/59 (69%,) I 12/31 (39%) C transforaminal interbody fusion 17/59 (29%) I,19/31 (61%) C Uninstrumented 1 (2%) I,0 (0%) C fusion levels 1: 36/59 (62%) I,20/31 (69%) C 2: 19/59 (32%) I,8/31 (27%) C 3: 4/59 (7%) I,3/31 (10%) C	**Intervention:** Cognitive behavioural therapy (CBT) – main topics included interaction of cognition & pain perception, coping strategies, pacing principles, ergonomic directions, return to work, & details about surgical procedure, 4wks, ×1/wk, 3hrs. Preoperative information about surgery, anaesthetic procedure, medication, postoperative rehabilitation, physical restrictions after surgery *.Post-surgery:* CBT refresher routine postoperative rehabilitation. **Usual care:** Preoperative information about surgery, anaesthetic procedure, medication, postoperative rehabilitation, physical restrictions after surgery routine postoperative rehabilitation	Psychological/behavioural (group) (supervised) Usual care (education) Psychological/behavioural (group) (supervised) Usual care (rehabilitation) Usual care (education) Usual care (rehabilitation)	A,Tr,R
Lindbäck 2018* [29] Fors 2019 [35] Sweden	197 randomised (99 I, 98 C) 169 pre-surgery FU (80 I, 89 C) 154 3 mth FU (72 I, 82C) 140 12 mth FU (62 I, 78 C) 197 analysed (99 I, 98 C) Female 54 (54%) I; 51 (52%) C Age, mean (SD) 58 yrs (13.3) I; 61 (11.5) C Spinal stenosis 59 (60%) I; 70 (71%) C Disc herniation 23 (23%) I; 17 (17%) C Spondylolisthesis 8 (8%) I; 7 (7%) C Degenerative disc disease 9 (9%) I; 4 (4%) C	**Intervention:** Spinal traction (TBC)^a^, 9 wks, ×2/wk;Specific movement (mechanical loading strategies incl. repeated movements according to directional preference) & mobilisation (TBC)^a^, 9wks, ×2/wk motor control exercises (trunk and pelvic floor muscle activation) (TBC)^a^, 9 wks, ×2/wk general exercise programme – 10 min intervals of CV exercise at beginning, middle & end of each session; 5–6 resistance and/or flexibility exercises based on patients’ function & posture, 9 wks, ×2/wk, >30 min Self-mediated home exercise & general physical activities, 9 wks, daily, 30 min; Behavioural approach –goal setting; strategies to minimise barriers to goal attainment (e.g., education regarding ergonomics, postural alignment, patho-anatomical/physiological explanation) standardised information about surgery, post surgery rehabilitation, and advice to stay active. *Post-surgery:* feedback on posture and walking, a home exercise program, and daily walking, which was followed up and progressed after 6 weeks. **Usual care:** standardised information about surgery, post surgery rehabilitation, and advice to stay active. *Post-surgery:* feedback on posture and walking, a home exercise program, and daily walking, which was followed up and progressed after 6 weeks.	Manual therapy^¥^ Manual therapy^¥^ Exercise (strength, trunk) (supervised) (individual) (not HEP) Exercise (CV, strength, flexibility) (supervised) (individual) (not HEP) Exercise (unsupervised) (individual) (HEP) Psychological/behavioural (supervised) (individual) Usual care (education) Usual care (rehabilitation) Usual care (education) Usual care (rehabilitation)	Tr,R Tr,R Tr,R Int,P, A,Tr A,Tr Tr
Marchand 2021 [25] Canada	68 randomised (35 I, 33 C) 55 pre-surgery FU (29 I, 26 C) 53 post-surgery FU (32 I, 21 C) 47 3 mth FU (24 I, 23 C) 43 6 mth FU (24 I, 19 C) 4 did not have surgery (1 I, 3 C) female, *n* (%): 14/35 (40%) I, 14/33 (42%) age, mean (SD): 66.2yrs (9.6) I,71.6 (7.6) C LSS Minimally invasive approach 11/34 (32%) I,8/30 (27%) C Open approach 23/34 (68%) I,22/30 (73%) C Operated vertebral segment(s) 1 level: 18 (53%) I,16 (53%) C 2 levels: 8 (24%) I,8 (27%) C 3 levels: 7 (20%) I,6 (20%) 4 levels: 1 (3%) I,0 (0%) C	**Intervention:** 5-min warm-up: cycling (stationary) or walking (treadmill);5 strength exercises with concentric or isometric phases (3×trunk stabilisation & posterior chain [squat, superman, leg raise]; 2×lower limb & hip [hips raise, hips abduction]), 6 wks, ×3/wk, 30 min routine hospital preoperative management; standardised written information on how to keep a good back posture when getting in or out of bed and when sitting down. **Usual care:** routine hospital preoperative management; standardised written information on how to keep a good back posture when getting in or out of bed and when sitting down.	Exercise (strength, trunk, lower limb) (supervised) (individual) (not HEP) Usual care (education) Usual care (education)	Int,P, A,R
Takenaka 2025 [26] Japan	32 randomised (15 I, 17 C), 28 baseline, post-prehab/presurgery FU/1/3mth post-surgery FU (13 I, 15 C), 27 6 mth FU (13 I, 14 C) Female, *n* (%): 8/13 (61.5%) I, 7/15 (46.7%) C Age, mean (SD) 69.7 (8.8) I,69.3 (7.7) C LSS Fusion 7/13 (53.8%) I,7/15 (46.7%) C Decompression 6/13 (46.2%) I,8/15 (53.3%) C Operation segments, *n* (%) 1: 6/13 (46.2%) I,9/15 (64.3%) C 2: 5/13 (38.5%) I,4/15 (28.6%) C 3: 2/13 (15.4%) I,0/15 C 4: 0/13 I,1/15 (7.1%) C1 C unaccounted for	**Intervention:** Education – LSS pathophysiology, perioperative timeline, recovery expectations + pamphlet Core strengthening exercise – 3 exercises, 2–3 times daily Walking programme – daily,duration based on individual symptoms Self-monitoring – daily log of exercise type, duration, and symptoms. Weekly progress review. **Usual Care:** Education – pamphlet containing the same information but no educational sessions	Education Exercise (HEP) (individual) (unsupervised) (strength, trunk)	A

*primary paper;I = intervention, C = control (usual care), FU = follow-up, TBC = treatment-based classification, LSS = lumbar spinal stenosis, ¥ =only 1 of 3 options for each participant depending on clasification,Int = exercise intensity described, P = exercise progression described, A = adherence strategies, Tr = clinician training described, R = rationale/justification for intervention described.

a Only one of three options for each participant depending on classification; ^b^ Only three participants involved; ^c^ No participants underwent alcohol abstinence programme.

Four of the five^26–29^ included trials had an overall risk of bias assessment of ‘some concerns’ and one^25^ had an overall assessment of ‘high’ due to the lack of blinding of the principal investigators (Supplementary information: C). All trials had ‘some concerns’ due to intervention assignment.

There were ‘some concerns’ for three studies due to missing data: Rolving^28^ had a high rate of missing data in one secondary outcome (back pain during first post-operative week). Lindback^29^ had a high rate of participant dropout at three months (22% overall, 27% prehabilitation group) and 12 months (29% overall, 37% prehabilitation group). Marchand^25^ also had a high post-surgical drop-out rate (26% overall, 29% prehabilitation group at three months; 33% overall, 29% prehabilitation group at six months).

Three studies exhibited ‘some concerns’^27,28,30^ and one trial^25^ was ‘high’ risk regarding outcome measurement due to issues with blinding of outcome assessors. For Rolving,^28^ outcome assessor blinding was a problem for one secondary outcome (Cumulated Ambulation Score first three post-operative days) whereas it is unclear how many outcomes may have been affected for the other three trials. Three studies demonstrated ‘some concerns’ in results reporting.^26,27,30^

Reporting quality varied with two of five trials describing 10 of 12 items,^25,28^ and the other three trials reporting six,^29^ three^26^ and one of 12 items^27^ of the Template for Intervention Description and Replication checklist adequately (Supplementary information: D). The remaining items were either partially reported or not reported at all. Only one item was universally reported (naming of intervention) with a further five generally reported across all five trials (procedure description, materials used, intervention setting, session frequency/duration, fidelity). Intervention delivery training, rationale, modifications and details about clinician experience were the items least well reported.

### Intervention design

Prehabilitation consisted of cognitive behavioural therapy,^28^ exercise alone,^25^ or exercise in combination with other components such as education,^26^ manual therapy, protein supplements and/or behavioural approaches.^27,29^ Two trials provided extra post-operative care for participants in the prehabilitation arm^27,28^ (Table 2).

Each intervention was broken down into a combined total of 47 individual components.

Four of the five trials included exercise as part of the prehabilitation programme, either alone or in combination with other modalities. The exercise components were either unsupervised home exercise programmes,^26,27^ or supervised individual programmes performed at a clinic or gym.^25,29^ One trial supplemented the supervised programme with 30 min/day of home exercise and general physical activity.^29^

Two trials included aerobic exercise to increase cardiovascular capacity,^27,29^ three trials included strength training, primarily targeting the trunk as well as the hip and lower limb.^25–27^^,29^ One trial reported flexibility exercises,^29^ and one trial included a daily walking regime.^26^

In addition to a specific exercise programme, Lindback^29^ prescribed motor control exercises for trunk and pelvic floor muscle activation to a subset of the participants in the prehabilitation group as part of their treatment-based classification protocol. The number of participants who received this was not reported.

Session duration ranged from 30 min^25,27^ up to 60 min^29^ per session with frequency ranging from once a day up to three times a day^26^ for home-based exercise, to three times a week for clinic/gym-based sessions.^25^ Duration of the exercise programme ranged from four to nine weeks. Only two of the four trials evaluating exercise provided some details regarding intensity and progression.^25,29^ Content, intensity, progression and volume/load was generally poorly described across studies.

Exercise programmes were delivered and/or supervised by kinesiologists,^25^ occupational therapists and/or physiotherapists^26,27,29^ and all clinical interactions were face-to-face (i.e., not online).

Four of the five included trials explicitly described psychological or behavioural components either alone^28^ or as part of the overall prehabilitation programme.^26,27,29^

Rolving^28^ delivered a four-week group cognitive behavioural therapy intervention which involved four three-hour pre-surgical sessions. Nielsen^27^ reported a six-week smoking cessation programme and a four-week alcohol abstinence programme for any smokers or ‘problem drinkers’ amongst their cohort, although only three smokers took part in the former and no participants undertook the latter.^31^ Lindback^29^ described using a behavioural approach in conjunction with a supervised exercise programme to reduce fear avoidance and maximise physical activity. Takenaka^26^ described the use of individual goal setting as part of the assessment.

In one of the five included trials, participants received one of three manual therapy techniques (i.e., mobilisation, spinal traction, or specific directional movement exercises to relieve symptoms) as part of a treatment-based classification approach. This was provided twice-a-week for nine weeks in conjunction with supervised exercise sessions.^29^ The number of participants who underwent each of these procedures was not reported.

Only one of the five included trials described a pre-surgical nutrition component^27^ which involved protein-rich supplements the evening prior to surgery. No mention was made by any trial of dietary advice.

All five trials provided education to all participants, usually including details of surgical and hospital procedures, post-operative processes and recovery. One trial included information on post-operative posture in sitting and activity.^25^

In addition to exercise, psychological/behavioural components and pre-surgical protein supplements, Nielsen^27^ provided an optimised analgesia regime to participants as part of the prehabilitation programme over and above that received by usual care participants.

In two of the five included trials, participants in the prehabilitation arm received extra post-operative care. Rolving^28^ provided a three-hour refresher cognitive behavioural therapy session at three- and six-months post-surgery. As part of participants’ in-patient stay, Nielsen^27^ provided four protein drink supplements/day, an optimised analgesia regime, follow-up visits for those on the smoking cessation programme, and double the amount of inpatient physiotherapy sessions. It is unclear as to the effect this may have had on outcomes measured after these time-points.

Three of the five included trials described a rationale or justification for the design of the prehabilitation programme.^25,26,28,29^ Only two trials described any training provided to the clinicians delivering the interventions.^28,29^

### Outcomes

Twenty-nine different outcomes were measured across the five trials at five common time points: post-prehabilitation/pre-surgery, perioperative, and post-surgery (three, six and 12 months) (Supplementary information: E). Rolving^28^ did not measure outcomes immediately after prehabilitation prior to surgery. Lindback^29^ did not measure six-month outcomes. Takenaka,^26^ Nielsen^27^ and Marchand^25^ did not measure 12-month outcomes. Results of the outcomes for each trial are described in Supplementary information: F-H. Data from Takenaka's pilot study^26^ was not used in the following syntheses as it was not powered sufficiently for hypothesis testing, therefore precluding any conclusions as to intervention effectiveness. This resulted in 28 outcomes used in subsequent syntheses.

For the primary synthesis, 16 of 28 outcomes showed greater improvement following prehabilitation compared to usual care though there were marked differences at each timepoint with the majority of improvement seen at the post-prehabilitation/pre-surgery timepoint (Tables 3 and 4). No difference between prehabilitation and control participants was reported for the remaining 12 outcomes. No outcome was reported to have worsened or improved to a lesser extent in comparison with controls as a result of prehabilitation.

**Table 3.** Summary study outcomes and result.

Outcome	Post-prehab/pre-surgery		Post-surgery				
			3 mth		6 mth		12 mth
Leg pain	ND^a,c^		ND^b,c^		ND^b,c^		ND^a,b^
Back pain	✓^a^	ND^c^	ND^b,c^		ND^b,c^		ND^a,b^
Disability	✓^a^	ND^c^	✓^b^	ND^a,c,d^	✓^b^	ND^c,d^	ND^a,b^
HRQoL	✓^a^	ND^d^	✓^b^	ND^a,d^	ND^b,d^		ND^a,b^
Fear avoidance	✓^a^	ND^c^	ND^b,c^		✓^b^	ND^c^	ND^a,b^
Depression	✓^a^	ND^c^	ND^a,c^		ND^c^		ND^a^
Self efficacy	✓^a^		ND^a^		Not evaluated		ND^a^
Anxiety	ND^a^		ND^a^		Not evaluated		ND^a^
Catastrophising	Not evaluated		ND^b^		✓^b^		ND^b^
Walking speed	✓^a^		Not evaluated		Not evaluated		Not evaluated
Walking time (time to first symptoms/total time)	✓^c^		Not evaluated		Not evaluated		Not evaluated
Sit-to-stand	ND^c,d^		ND^d^		ND^d^		Not evaluated
Timed-up-and-go	ND^c,d^		ND^d^		ND^d^		Not evaluated
Physical activity (self report)	✓^a^		Not evaluated		Not evaluated		✓^a^
Walking distance (self-report)	✓^a^		Not evaluated		Not evaluated		Not evaluated
Global impression of change	✓^a,c^		*Not reported*		Not evaluated		*Not reported*

✓ = greater improvement in favour of prehabilitation; ND = no between-group difference.

a Lindback (*n* = 197) ^b^ Rolving (*n* = 96) ^c^ Marchand (*n* = 68) ^d^ Nielsen (*n* = 73).

**Table 4.** Summary study outcomes and result.

Outcome	Perioperative/post-surgery	
Hospital stay	✓^d^	ND^b,c^
Inpatient mobility milestones	✓^b,d^	
Satisfaction (overall treatment & outcome 1 mth post-op)	✓^d^	
Satisfaction (effect of surgery on leg/back pain)	ND^c^	
Adverse events	ND^d^	
Analgesia (1^st^ week post-op/12 mths post-op)	ND^b^	
Complications	ND^d^	
Return to work	ND^b,d^	
Health resource use	ND^b^	
Walking time (time to first symptoms/total time at 6 wks)	ND^c^	
Sit-to-stand (at 4–6 wks)	ND^c,d^	
Timed-up-and-go (at 4–6 wks)	ND^c,d^	
LBP 1^st^ week post-op	ND^b^	

✓ = greater improvement in favour of prehabilitation; ND = no between-group difference.

a Lindback (*n* = 197); ^b^ Rolving (*n* = 96); ^c^ Marchand (*n* = 68); ^d^ Nielsen (*n* = 73).

**Figure 2.**
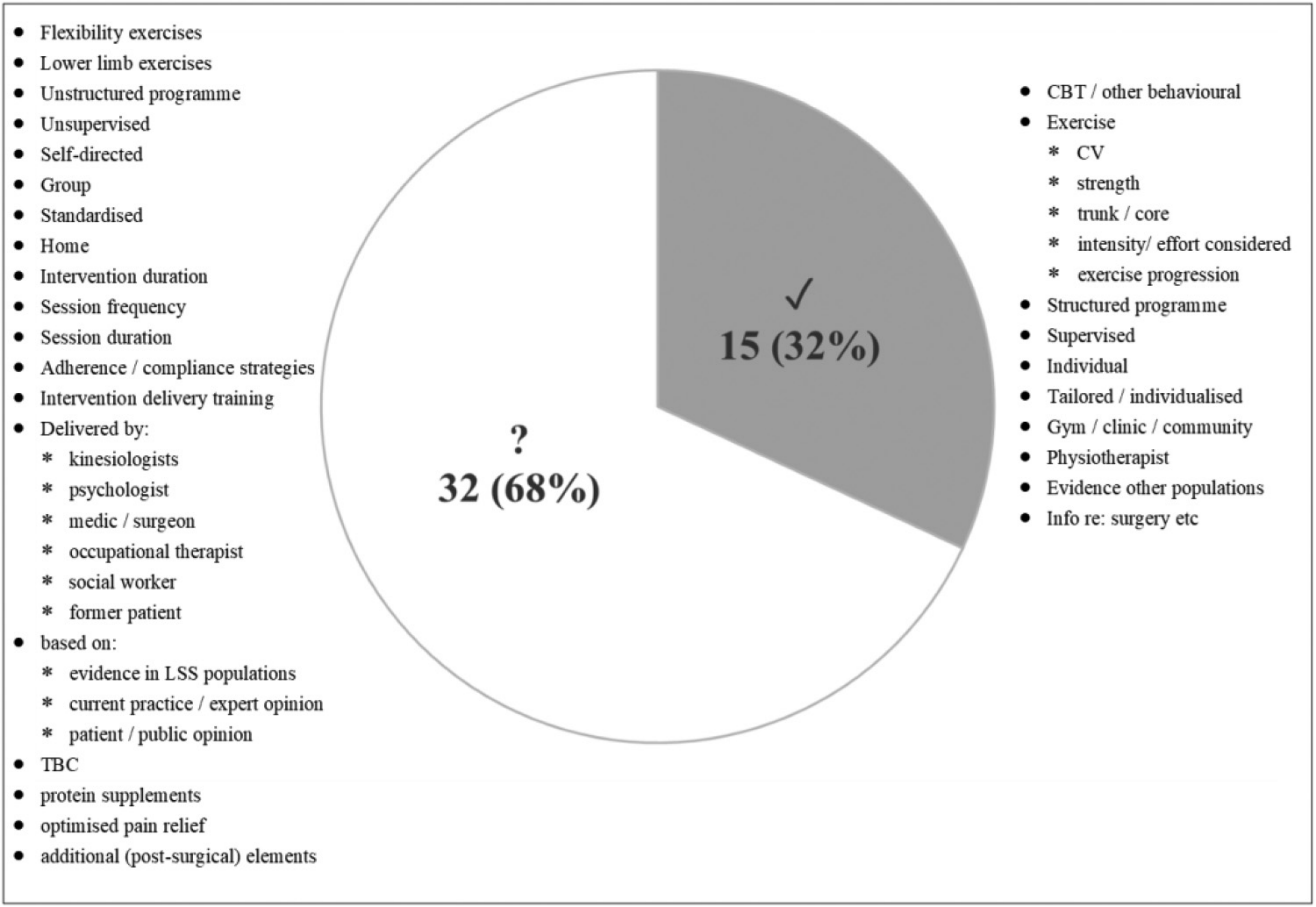
Intervention components featuring in >50% (✓) and ≤50% (?) of successful trial interventions (overall analysis with all outcomes at all timepoints).

Mapping of the 47 identified individual components from the four trial interventions across all timepoints (post-prehabilitation/pre-surgery, perioperative, post-surgery) and all 28 outcomes revealed that 15/47 individual components featured in successful interventions at some timepoint with at least one outcome measure. The remaining 32/47 components did not reach the threshold criteria to be classified as part of unsuccessful or harmful interventions (Table 5) (Figure 2).

**Table 5.** Summary of components featured in successful interventions – overall (all timepoints and outcomes).

Component	Nielsen (*n* = 73)^c^	Rolving^a,c^ (*n* = 96)	Lindback (*n* = 197)	Marchand (*n* = 68)	Frequency component featured in successful intervention/*n* (studies with successful interventions)
CBT/other behavioural	✓	✓	✓	** -**	3/4
Exercise	✓	-	✓	✓	3/4
- CV	✓	NA	✓	** -**	2/3*
- strength	✓	NA	✓	✓	3/3*
- trunk/core	✓	NA	NR	✓	2/3*
- intensity/effort considered	NR	NA	✓	✓	2/3*
- exercise progression	NR	NA	✓	✓	2/3*
Structured programme	✓	✓	✓	✓	4/4
Supervised	** -**	✓	✓	✓	3/4
Individual	✓	** -**	✓	✓	3/4
Tailored/individualised	✓	**-**	✓	✓	3/4
Gym/clinic/community	** -**	✓	✓	✓	3/4
Physiotherapist delivered	✓	✓	✓	** -**	3/4
Evidence other populations	-	✓	✓ (TBC)^b^	✓	3/4
Info re: surgery etc	✓	✓	✓	✓	4/4

✓ **=** featured in successful intervention; NA = not applicable; NR = not reported. * Rolving did not include exercise so only three studies looking at exercise-specific components.

a Rolving did not record post-prehab/pre-surgery outcomes.

b TBC – treatment-based classification – 1 of either traction, pelvic floor/trunk motor control exercises or specific symptom reducing directional movements.

c Rolving – 3 hr CBT refresher session at 3 and 6 mths; Nielsen – double physio inpatient rehab time, optimised pain regime, protein supplements, smoking cessation follow-up.

Outcomes that improved to greater extent as a result of prehabilitation compared to usual care:

Lindback – post-prehab: back pain, disability, HRQoL, fear avoidance, depression, self-efficacy, self-reported walking distance, walking speed, global impression of change; 12 mths: self-reported physical activity.

Rolving – perioperative: inpatient mobility milestones; 3 mths: disability, HRQoL; 6 mths: disability, fear avoidance, catastrophising.

Marchand – post-prehab: global impression of change, walking time to first symptoms, total walking time.

Nielsen – perioperative: hospital stay, inpatient mobility milestones, satisfaction.

Exercise and psychological/behavioural components featured in three of four successful interventions; a structured intervention programme in all (4/4) successful interventions; cardiovascular, trunk/core exercises and strength also featured in two, two and three of three successful exercise interventions respectively along with supervised programmes that treated participants individually rather than in groups, that were tailored/individualised and took place in a clinic or community setting (all featured in three of four successful interventions). Exercise intensity and progression protocols were described in two of three successful exercise interventions and they were designed in accordance with evidence from populations with other related conditions (three of four interventions). Information regarding surgery and post-operative recovery etc was provided in all four successful interventions and the programmes were delivered by physiotherapists in three of four successful interventions.

There was no consensus on intervention duration, frequency of sessions, or individual session duration.

Only three outcomes were measured by more than two studies at the same timepoint (Supplementary information: E). Secondary syntheses were therefore undertaken for the following outcomes: (1) disability: pre-surgery, three- and six-months post-surgery; (2) health-related quality of life: three months post-surgery; (3) hospital stay.

However, these syntheses proved unreliable, with inconsistent and, at times, contradictory results. The results are not included within the main report but data tables for each of these secondary syntheses are provided in Supplementary information: I-M.

## Discussion

Five randomised controlled trials of prehabilitation for lumbar spinal stenosis and related conditions were identified, although, due to the small number of participants, one study was not included in the syntheses. Overall, 15 components featured in interventions resulting in greater improvement in prehabilitation compared to control participants. No trial interventions were deemed unsuccessful or harmful, therefore identifying components to exclude from future prehabilitation interventions was not possible.

However, the paucity of trials specific to lumbar spinal stenosis resulted in the inclusion of trials with other related lumbar spinal conditions and the sample sizes of the included trials were generally small, reducing the certainty of their findings. In particular, Marchand^25^ did not recruit to target and was underpowered to detect between group differences. Along with Lindback,^29^ they reported a high drop-out rate particularly at later follow-up timepoints.

The included trials also involved prehabilitation interventions of relatively short duration. In comparison to the time potentially spent waiting for surgery in the UK, this is a particularly short period. Prehabilitation also tends to be concentrated immediately prior to surgery which could result in people waiting for a considerable time with consequent deterioration before they access any treatment. Only two trials measured outcomes beyond six months after surgery, reducing the ability to evaluate longer term effects.^28,29^

Several systematic reviews have explored prehabilitation for a variety of conditions including spinal conditions. McIsaac et al.'s^13^ review of prehabilitation for people undergoing surgery concluded that there were consistent and potentially meaningful benefits of combined interventions, including exercise and nutritional components, although further high quality and well-powered trials are required to have greater certainty in their efficacy. However, overall the evidence is conflicting with Janssen's^32^ review of cognitive behavioural therapy prehabilitation for patients awaiting spinal surgery concluding with low-certainty evidence that prehabilitation does not have any added benefit for postoperative outcomes. A further meta-analysis of prehabilitation for patients undergoing elective spinal surgery reported inconsistent evidence with low quality of evidence for post-operative outcomes.^16^ These systematic reviews and meta-analyses generally confirm our findings and highlight the lack of evidence either for or against prehabilitation, particularly in relation to lumbar spinal stenosis. However, due to the nature of their analyses, they were unable to determine which, if any, components were associated with successful interventions.

One of the strengths of this review is the comprehensive search strategy and the inclusion of only randomised controlled trials, providing greater confidence that any between group differences could be attributed to the intervention, as well as in the identification of individual components associated with successful interventions. The Intervention Component Analysis approach has been demonstrated to provide in-depth information regarding differences between trial interventions, allowing a more detailed investigation of individual components and their importance to trial outcomes. Assessment of reporting and risk of bias using recognised tools provides further information regarding the quality of the included trials. Two reviewers, a priori registration of the protocol and adherence to the Preferred Reporting Items for Systematic Reviews and Meta-Analyses reporting guidelines are also strengths.

However, only English language papers were included and no searches were conducted of unpublished or ‘grey’ literature. In identifying intervention components, the small number of trials resulted in extremes where the addition or subtraction of one trial could easily alter the result. We therefore cannot be confident in our assessment of the association between intervention components and outcome results in general, and particularly in subgroup syntheses related to individual outcome measures or timepoints. We have identified components to be considered when designing prehabilitation programmes, but the results of this review are highly sensitive to any new evidence.

Use of a 50% threshold for categorising intervention components lacks evidence to support its use. Comer^22^ used a 75% threshold in their analysis of exercise interventions for lumbar spinal stenosis. However, the 13 trials in their review resulted in more trial interventions available for comparison and reduced variability in their syntheses. The review could only consider intervention components that featured in the four included trials and no conclusions can be drawn as to the usefulness of other potential components. Some of the reported between-group differences between prehabilitation and control participants may not be clinically relevant. This could compromise the decisions made regarding whether interventions resulted in successful treatment effects. Any between-group differences in outcomes also tended to be short term and very few endured post-surgery. In addition, two of the trials involved intensive post-surgery components for prehabilitation participants that may have had an unquantifiable impact on post-surgery outcomes.^27,28^

In conclusion, this review aimed to identify the effect of prehabilitation interventions and to identify individual intervention components that lead to improved outcomes in people undergoing surgery for lumbar spinal stenosis and related degenerative spinal conditions. Overall, 15 components were identified as featuring in prehabilitation interventions that resulted in greater improvement in outcomes compared to usual care. However, due to the small number of trials, the small numbers of participants involved and issues with individual trials, further in-depth analysis was problematic and confidence in these results is low. It is highly likely that they will change with new evidence.

**Clinical Messages**

People undergoing prehabilitation for lumbar spinal stenosis surgery:

- gain small benefits in some outcomes that generally occur pre-surgery rather than post-surgery.
- the evidence is from a few, small, low-to-moderate quality trials.
- although some components associated with successful interventions were identified that could be considered when designing a prehabilitation programme, more evidence is needed for greater certainty and precision regarding the effect on individual outcomes at different timepoints.

## Supplemental Material

sj-docx-1-cre-10.1177_02692155261418206 - Supplemental material for The effectiveness of prehabilitation on post-operative recovery from lumbar spinal stenosis surgery – A systematic review and intervention component analysis Supplemental material, sj-docx-1-cre-10.1177_02692155261418206 for The effectiveness of prehabilitation on post-operative recovery from lumbar spinal stenosis surgery – A systematic review and intervention component analysis by Peter Heine, Rebecca Hunter, Andrew Booth, Sarah E Lamb, Esther Williamson, Opinder Sahota, Bethan E Phillips, Paul Hendrick and Lianne Wood in Clinical Rehabilitation
