# Supplementary material for: The effectiveness of prehabilitation on post-operative recovery from lumbar spinal stenosis surgery – A systematic review and intervention component analysis
Source: Clin Rehabil. 2026 Mar 14;40(7):906–23. doi: 10.1177/02692155261418206 (PMC13283504; doi:10.1177/02692155261418206)
Supplement: sj-docx-1-cre-10.1177_02692155261418206 - Supplemental material for The effectiveness of prehabilitation on post-operative recovery from lumbar spinal stenosis surgery – A systematic review and intervention component analysis [file sj-docx-1-cre-10.1177_02692155261418206.docx]

**List of Supplementary Information:**

A. Search strategy

B. Additional methods for how the intervention component analysis was performed

C Risk of bias for included studies

D. Quality of intervention reporting – TIDieR checklist

E. Number of trials that measured each outcome (by timepoint)

F. Summary of study results - self report outcomes

G. Summary of study results - objective outcomes

H. Summary of study results – perioperative outcomes

I. Summary of components featured in successful interventions – disability (post-prehabilitation / pre-surgery)

J. Summary of components featured in successful interventions – disability (3 months)

K. Summary of components featured in successful interventions – disability (6 months)

L. Summary of components featured in successful interventions – HRQoL (3 months)

M. Summary of components featured in successful interventions – hospital stay

A. Search strategy

| Source | Date searched | Search strategy |
| --- | --- | --- |
| PubMed | 22/01/2025 | Search Actions Details Query Results Time  #16 Search: #14 NOT #10 Filters: from 2024/6/1 - 2025/1/31 Sort by: Most Recent 1,167  #14 Search: #11 OR #12 OR #13 Filters: from 2024/6/1 - 2025/1/31 Sort by: Most Recent 1,221  #13 Search: "preoperative rehabilitation" Filters: from 2024/6/1 - 2025/1/31 Sort by: Most Recent 21  #12 Search: preoperative rehabilitation Filters: from 2024/6/1 - 2025/1/31 Sort by: Most Recent 1,049  #11 Search: prehabilitation[tiab] Filters: from 2024/6/1 - 2025/1/31 Sort by: Most Recent 351  #10 Search: #5 AND #6 Filters: from 2024/6/1 - 2025/1/31 Sort by: Most Recent 54  #6 Search: prehabilitation Sort by: Most Recent 6,647  #5 Search: Orthopedic Procedures Sort by: Most Recent 392,596 |
| CINAHL | 22/01/2025 | S3 (prehabilitation or prehab or pre-operative rehabilitation or peri-operative rehabilitation) AND ((MH "Spinal Diseases+") OR (MH "Orthopedic Surgery+")) |
| Scopus | 22/01/2025 | ( ALL ( prehabilitation OR "preoperative rehabilitation" OR "pre-operative rehabilitation" ) AND TITLE-ABS-KEY ( lumbar OR spine OR spinal OR orthoped* OR orthopaed* ) ) AND PUBYEAR > 2023 AND PUBYEAR < 2026 AND ( LIMIT-TO ( SUBJAREA , "MEDI" ) OR LIMIT-TO ( SUBJAREA , "HEAL" ) OR LIMIT-TO ( SUBJAREA , "NURS" ) ) AND ( LIMIT-TO ( LANGUAGE , "English" ) ) AND ( LIMIT-TO ( EXACTKEYWORD , "Human" ) ) |
| Web of Science | 22/01/2025 | (TS=(prehabilitation OR "Preoperative rehabilitation" OR "pre operative rehabilitation" )) AND TS=(Orthopaed* Or Orthoped* Or Lumbar Or Spinal Or Spine. )  Publication Date: 2024-06-01 to 2025-01-31 |

B. Additional methods for how the intervention component analysis was performed

The components of each trial intervention were tabulated along with the context, setting, delivery method, frequency, duration and other relevant intervention details. Standardisation across interventions was introduced by grouping similar components together (e.g. smoking cessation programmes, cognitive behavioural therapy into ‘psychological/behavioural’ components). The presence of adherence strategies, clinician training and rationale or justification for the intervention design were also included. The components were then cross-checked with the outcomes of each study to determine whether they featured in interventions associated with an outcome that reported between group differences at each time-point.

Interventions were categorised as:

1. ‘successful’ - resulted in a statistically significant between group difference in relevant outcomes in favour of prehabilitation compared to control participants.

2. ‘unsuccessful’ - did not result in a statistically significant between group difference.

3. ‘harmful’ - resulted in a statistically significant between group difference in favour of control compared to prehabilitation participants.

Individual intervention components were then evaluated using a threshold to determine whether individual components were typically involved in successful, unsuccessful or harmful interventions. To be considered a consistent element of successful interventions, components needed to feature in >50% of successful interventions and ≤50% of unsuccessful/harmful interventions for the outcome(s) in question. Components that featured in >50% of unsuccessful interventions and ≤50% of successful/harmful studies were consistent elements of unsuccessful interventions. Harmful components needed to feature in >50% of harmful and ≤50% of successful/unsuccessful interventions. Any components not captured by these threshold criteria did not feature consistently in any single intervention type.

We planned two types of syntheses. Firstly, intervention components were mapped onto all available outcomes and timepoints to obtain an overall summary. Secondly, syntheses we attempted to explore in greater detail by mapping intervention components onto individual outcomes at each timepoint.

C. Risk of bias for included studies

| study | | Randomisation | Intervention assignment | Missing data | Outcome measurement | Results reporting | Overall |
| --- | --- | --- | --- | --- | --- | --- | --- |
| Nielsen | 2008 | Low | Some concerns | Low | Some concerns | Some concerns | Some concerns |
|  | 2010 | Low | Some concerns | Low | Some concerns | Some concerns | Some concerns |
| Rolving | 2015 | Low | Some concerns | Low | Low | Low | Some concerns |
|  | 2016 | Low | Some concerns | Some concerns | Some concerns | Low | Some concerns |
|  | 2016b | Low | Some concerns | Low | Low | Low | Some concerns |
| Lindback | 2018 | Low | Some concerns | Some concerns | Low | Low | Some concerns |
| Fors | 2019 | Low | Some concerns | Low | Low | Low | Some concerns |
| Marchand | 2021 | Low | Some concerns | Some concerns | High | Low | High |
| Takenaka | 2025 | Low | Some concerns | Low | Low | Some concerns | Some concerns |

D. Quality of intervention reporting – TIDieR checklist

|  | Brief name | Why | What materials | What procedures | Who | How | Where | When & how much | Tailoring | Modifications | How well planned | Actual treatment fidelity |
| --- | --- | --- | --- | --- | --- | --- | --- | --- | --- | --- | --- | --- |
| Nielsen 2008, 2010 | YES | Not described | Partially | Partially | Not described | Not described | Partially | Partially | Not described | not reported | Partially | Partially |
| Rolving 2015, 2016, 2016b | YES | YES | YES | YES | YES | YES | YES | YES | YES | not reported | Partially | YES |
| Lindbäck 2018 Fors 2019 | YES | Partially | Partially | YES | Partially | Partially | YES | YES | Partially | not reported | YES | YES |
| Marchand 2021 | YES | YES | YES | YES | Partially | YES | YES | YES | YES | not reported | YES | YES |
| Takenaka 2025 | YES | Not described | YES | YES | Partially | Partially | Partially | Partially | Partially | not reported | Partially | Not described |

E. Number of trials that measured each outcome (by timepoint)

| outcome | post-prehab /  pre-surgery | perioperative | post-surgery | | | *max number of studies / outcome* |
| --- | --- | --- | --- | --- | --- | --- |
|  |  |  | 3 mths | 6 mths | 12mths |  |
| leg pain | 2 | 0 | 2 | 2 | 2 | *2* |
| back pain | 2 | 0 | 2 | 2 | 2 | *2* |
| **disability** | **3** | 0 | **4** | **3** | 2 | ***4*** |
| **HRQoL** | 2 | 0 | **3** | 2 | 2 | ***3*** |
| fear avoidance | 2 | 0 | 2 | 2 | 2 | *2* |
| depression | 2 | 0 | 2 | 1 | 1 | *2* |
| anxiety | 1 | 0 | 1 | 0 | 1 | *1* |
| self-efficacy | 1 | 0 | 1 | 0 | 1 | *1* |
| catastrophising | 0 | 0 | 1 | 1 | 1 | *1* |
| physical activity (self-reported) | 1 | 0 | 0 | 0 | 1 | *1* |
| walking distance (self-reported) | 1 | 0 | 0 | 0 | 0 | *1* |
| walking speed | 1 | 0 | 0 | 0 | 0 | *1* |
| walking time to first symptoms | 1 | 1 | 0 | 0 | 0 | *1* |
| walking time (total) | 1 | 1 | 0 | 0 | 0 | *1* |
| sit-to-stand | 2 | 2 | 1 | 1 | 0 | *2* |
| timed-up-and-go | 2 | 2 | 1 | 1 | 0 | *2* |
| **hospital stay** | NA | **3** | NA | NA | NA | ***3*** |
| adverse events | NR | 1 | NR | NR | NR | *1* |
| analgesia | NR | 1 | NR | NR | 1 | *1* |
| complications | NR | 1 | NR | NR | NR | *1* |
| return to work | NA | 1 | NR | NR | 1 | *1* |
| sick leave | NR | NA | NR | NR | 1 | *1* |
| healthcare resource use | NR | NA | NR | NR | 1 | *1* |
| inpatient mobility | NA | 2 | NA | NA | NA | *2* |
| LBP 1^st^ week post-op | NA | 1 | NA | NA | NA | *1* |
| change in condition | 2 | NR | NR | NR | NR | *2* |
| satisfaction | NR | 2 | NR | NR | NR | *2* |
| NA = not applicable; NR = not reported | | | | | | |

F. Summary of study results - self report outcomes

|  | **measurement**  **tool** | **Intervention** | **Control** | **between group change** | **p-value** | **comments** |
| --- | --- | --- | --- | --- | --- | --- |
| **Leg Pain** |  |  |  |  |  |  |
| **post-prehab / pre-surgery** | | | | | | |
| Lindback 2018  n=197^1^ | VAS 0-100 (0 - 100 worst) | Mean (SE) change from baseline [effect size, Cohen d] -10.5 (2.5), [-0.5], n=99 | Mean (SE) change from baseline [effect size, Cohen d] -5.0 (2.5), [-0.2], n=98 | Adjusted for baseline score Between group, mean change (95%CI) -4.6 (-10.8 to 1.5), n=197 | between group change (adjusted scores) p=0.140 |  |
| Marchand 2021  n=55 | 11 point NRS (0 - 10 worst) | mean ± SD (95% CI)  5.5 ± 2.3 (4.6 to 6.4), n=29 | mean ± SD (95% CI) 6.5 ± 2.4 (5.5 to 7.4), n=26 | - | p=0.76 (main effect of group) p=0.03 (group x time interaction) |  |
| **short term post-surgery (3mths)** | | | | | | |
| Rolving 2015  n=80 | 11 point NRS (0 - 10 worst) | median (IQR) change score -3.2 (-5.3, -1.3), n = 54 | median (IQR) change score -2.3 (-4.7, -0.3), n = 26 | - | between group change p=0.23 |  |
| Marchand 2021  n=47 | 11 point NRS (0 - 10 worst) | mean ± SD (95% CI) 2.9 ± 3.1 (1.6 to 4.3), n=24 | mean ± SD (95% CI) 2.2 ± 2.3 (1.2 to 3.2), n=23 | - | p=0.99 (main effect of group) p=0.30 (group x time interaction)^2^ |  |
| **medium term post-surgery (6mths)** | | | | | | |
| Rolving 2015  n=80 | 11 point NRS (0 - 10 worst) | median (IQR) change score -2.8 (-5.0, -1.3), n = 55 | median (IQR) change score -2.0 (-5.7, -0.3), n = 25 |  | between group change p=0.43 |  |
| Marchand 2021  n=43 | 11 point NRS (0 - 10 worst) | mean ± SD (95% CI) 2.4 ± 2.8 (1.2 to 3.6), n=24 | mean ± SD (95% CI) 3.1 ± 2.5 (1.8 to 4.3), n=19 | - | p=0.99 (main effect of group) p=0.30 (group x time interaction)^2^ |  |
| **long term post-surgery (12mths)** | | | | | | |
| Rolving 2015  n=81 | 11 point NRS (0 - 10 worst) | median (IQR) change score -2.8 (-4.7, -1.3), n = 54 | median (IQR) change score -1.3 (-6.0, -0.3)), n = 27 | - | between group change p=0.70 |  |
| Lindback 2018  n=197^1^ | VAS 0-100 (0 - 100 worst) | Mean (SE) change from baseline [effect size, Cohen d] -35.0 (3.3), [-1.4], n=99 | Mean (SE) change from baseline [effect size, Cohen d] -36.5 (3.3), [-1.4], n=98 | Adjusted for baseline score Between group, mean change (95%CI) 2.9 (−5.4 to 11.2), n=197 | between group change (adjusted scores) p=0.484 |  |
| **Low Back Pain** | | | | | | |
| **post-prehab / pre-surgery** | | | | | | |
| Lindback 2018  n=197^1^ | VAS 0-100 (0 - 100 worst) | Mean (SE) change from baseline [effect size, Cohen d] −7.9 (2.3), [−0.5], n=99 | Mean (SE) change from baseline [effect size, Cohen d] −3.4 (2.3), [−0.2], n=98 | **Adjusted for baseline score Between group, mean change (95%CI) -6.0 (-11.8 to -0.3), n=197*** | **between group change (adjusted scores) p=0.040*** |  |
| Marchand 2021  n=55 | 11 point NRS (0 - 10 worst) | mean ± SD (95% CI) 4.5 ± 2.2 (3.6 to 5.3), n=29 | mean ± SD (95% CI) 5.0 ± 2.7 (3.9 to 6.1), n=26 | - | p=0.69 (main effect of group) p=0.91 (group x time interaction) |  |
| **short term post-surgery (3mths)** | | | | | | |
| Rolving 2015  n=80 | 11 point NRS (0 - 10 worst) | median (IQR) change score -3.0 (-4.3, -1.3), n = 54 | median (IQR) change score -2.6 (-4.3, -0.3), n = 26 | - | between group change p=0.41 |  |
| Marchand 2021  n=47 | 11 point NRS (0 - 10 worst) | mean ± SD (95% CI) 1.6 ± 1.7 (0.9 to 2.3), n=24 | mean ± SD (95% CI) 2.6 ± 2.4 (1.5 to 3.6), n=23 | - | p=0.17 (main effect of group) p=0.90 (group x time interaction)^2^ |  |
| **medium term post-surgery (6mths)** | | | | | | |
| Rolving 2015  n=80 | 11 point NRS (0 - 10 worst) | median (IQR) change score -2.3 (-4.0, -1.7), n = 55 | median (IQR) change score -2.3 (-4.7, -0.7), n = 25 |  | between group change p=0.94 |  |
| Marchand 2021  n=43 | 11 point NRS (0 - 10 worst) | mean ± SD (95% CI) 2.4 ± 2.6 (1.3 to 3.5), n=24 | mean ± SD (95% CI) 3.7 ± 2.6 (2.4 to 4.9), n=19 | - | p=0.17 (main effect of group) p=0.90 (group x time interaction)^2^ |  |
| **long term post-surgery (12mths)** | | | | | | |
| Rolving 2015  n=81 | 11 point NRS (0 - 10 worst) | median (IQR) change score -2.5 (-4.3, -1.0), n = 54 | median (IQR) change score -2.7 (-5.0, -0.3), n = 27 | - | between group change p=0.81 |  |
| Lindback 2018  n=197^1^ | VAS 0-100 (0 - 100 worst) | Mean (SE) change from baseline [effect size, Cohen d] -24.5 (3.0), [−1.5], n=99 | Mean (SE) change from baseline [effect size, Cohen d] -31.8 (3.0), [−1.9], n=98 | Adjusted for baseline score Between group change mean change (95%CI) 4.7 (−2.4 to 11.8), n=197 | between group change (adjusted scores) p=0.195 |  |
| **Disability** |  |  |  |  |  |  |
| **post-prehab / pre-surgery** | | | | | | |
| Neilson 2010  n=60 | Roland Morris Questionnaire (0 - 24 worst) | median (range) 14 (1–21), n=28 | median (range) 17 (7–23), n=32 | - | p=0.001? | unclear if is between or within group difference |
| Lindback 2018  n=197^1^ | Oswestry Disability Index (0 -100 worst) | Mean (SE) change from baseline [effect size, Cohen d] -3.2 (1.1), [-0.3], n=99 | Mean (SE) change from baseline [effect size, Cohen d] -0.6 (1.1), [0.0], n=98 | **Adjusted for baseline score Between group, mean change (95%CI) -3.3 (-6.2 to -0.4), n=197*** | **between group change (adjusted scores) p=0.027*** |  |
| Marchand 2021  n=55 | Oswestry Disability Index (0 -100 worst) | mean ± SD (95% CI) 35.3 ± 17.4 (28.7 to 41.9), n=29 | mean ± SD (95% CI) 39.5 ± 14.9 (33.5 to 45.5), n=26 | - | p=0.29 (main effect of group) p=0.12 (group x time interaction) |  |
| Marchand 2021  n=17 | French Swiss Spinal Stenosis Questionnaire (12 best -55 worst) | Mean (SE) change from baseline [effect size, Cohen d] 35.0 ± 7.9 (28.9 to 41.0), n=9 | Mean (SE) change from baseline [effect size, Cohen d] 37.5 ± 6.5 (32.1 to 42.9), n=8 | - | p=0.47 (main effect of group) p=0.004 (group x time interaction) | scores out of 55 for preop and 79 post-op |
| **short term post-surgery (3mths)** | | | | | | |
| Neilson 2010  n=56 | Roland Morris Questionnaire (0 - 24 worst) | median (range) 8 (0–20), n=28 | median (range) 11 (0–22), n=28 | - | no reported difference |  |
| Rolving 2015 2016b^3^  n=81 | Oswestry Disability Index (0 -100 worst) | **median (IQR) change score -15 (-26, -4), n = 55*** | **median (IQR) change score 1 (-14, 8), n = 26*** | **Between group, mean change (95% CI) -10.8 (17.8, -3.8), n=81*** | **between group difference p=0.003*** | Between group mean change from 2016b paper; reported mean (95% CI) change score rather than median (IQR) score, p=0.003* |
| Lindback 2018  n=197^1^ | Oswestry Disability Index (0 -100 worst) | Mean (SE) change from baseline [effect size, Cohen d] -10.6 (1.7), [-1.0], n=99 | Mean (SE) change from baseline [effect size, Cohen d] -14.0 (1.7), [-1.3], n=98 | Adjusted for baseline score Between group, mean change (95%CI) 1.6 (-2.9 to 6.1), n=197 | between group change (adjusted scores) p=0.495 |  |
| Marchand 2021  n=47 | Oswestry Disability Index (0 -100 worst) | mean ± SD (95% CI) 15.8 ± 13.6 (10.1 to 21.6), n=24 | mean ± SD (95% CI) 22.0 ± 13.9 (15.9 to 28.0), n=23 | - | p=0.36 (main effect of group) p=0.003 (group x time interaction)^2^ |  |
| Marchand 2021x  n=14 | French Swiss Spinal Stenosis Questionnaire (18 best -79 worst) | mean ± SD (95% CI) 37.9 ± 10.2 (29.3 to 46.4), n=8 | mean ± SD (95% CI) 39.2 ± 14.4 (24.0 to 54.3), n=6 | - | p=0.72 (main effect of group) p=0.83 (group x time interaction)^2^ | scores out of 55 for preop and 79 post-op |
| **medium term post-surgery (6mths)** | | | | | | |
| Neilson 2010  n=56 | Roland Morris Questionnaire (0 - 24 worst) | median (range) 8 (0–20), n=28 | median (range) 11 (0–23), n=28 | - | no reported difference |  |
| Rolving 2015 2016b^3,4^  n=80 | Oswestry Disability Index (0 -100 worst) | median (IQR) change score -18 (-24, -7), n = 55 | median (IQR) change score -4 (-16, 4), n = 25 | **Between group, mean change (95% CI) -6.8 (-0.8, -13.4), n=80*** | between group median difference p=0.056 | **Between group mean change p=0.047*** |
| Marchand 2021  n=43 | Oswestry Disability Index (0 -100 worst) | mean ± SD (95% CI) 12.7 ± 16.0 (6.1 to 19.4), n=24 | mean ± SD (95% CI) 24.3 ± 14.3 (17.6 to 31.0), n=19 | - | p=0.36 (main effect of group) p=0.003 (group x time interaction)^2^ |  |
| Marchand 2021  n=12 | French Swiss Spinal Stenosis Questionnaire (18 best -79 worst) | Mean (SE) change from baseline [effect size, Cohen d] 30.5 ± 10.1 (22.7-38.4), n=9 | Mean (SE) change from baseline [effect size, Cohen d] 45.0 ± 21.6 (8.8-78.8), n=3 | - | p=0.72 (main effect of group) p=0.83 (group x time interaction)^2^ | scores out of 55 for preop and 79 post-op |
| **long term post-surgery (12mths)** | | | | | | |
| Rolving 2015 2106b^3^  n=85 | Oswestry Disability Index (0 -100 worst) | median (IQR) change score -14 (-26, -5), n=56 | median (IQR) change score -6 (-26, 4), n=29 | Between group, mean change (95% CI) -5.0 (-11.6, 1.7), n=85 | between group difference p=0.082 | Between group mean change p=0.14 |
| Lindback 2018  n=197^1^ | Oswestry Disability Index (0 -100 worst) | Mean (SE) change from baseline [effect size, Cohen d] -15.0 (1.7), [-1.4], n=99 | Mean (SE) change from baseline [effect size, Cohen d] -20.4 (1.7), [-1.9], n=98 | Adjusted for baseline score Between group change, mean diff (95%CI) 4.0 (-0.5 to 8.5), n=197 | between group change (adjusted scores) p=0.080 |  |
| **HRQoL** |  |  |  |  |  |  |
| **post-prehab / pre-surgery** | | | | | | |
| Nielsen 2010  n=60 | Quality of life survey 15D (0 worst - 1 best) | median (range) 0.85 (0.74–0.99), n=28 | median (range) 0.82 (0.65–0.92), n=32 | - | no reported difference |  |
| Lindback 2018  n=197^1^ | EQ5D (-0.594 worst - 1 best) | Mean (SE) change from baseline [effect size, Cohen d] 0.121 (0.0), [0.4], n=99 | Mean (SE) change from baseline [effect size, Cohen d] 0.054 (0.0), [0.2], n=98 | **Adjusted for baseline score Between group, mean change (95%CI) 0.078 (0.0 to 0.1), n=197*** | **between group change (adjusted scores) p=0.027*** |  |
| Lindback 2018  n=197^1^ | Short Form (SF-36) Physical Component Score (0 - 100 best) | Mean (SE) change from baseline [effect size, Cohen d] 2.4 (0.7), [0.3], n=99 | Mean (SE) change from baseline [effect size, Cohen d] 1.6 (0.7), [0.2], n=98 | Adjusted for baseline score Between group, mean change (95%CI) -1.5 (−0.5 to 3.5), n=197 | between group change (adjusted scores) p=0.129 |  |
| Lindback 2018  n=197^1^ | Short Form (SF-36) Mental Component Score (0 - 100 best) | Mean (SE) change from baseline [effect size, Cohen d] 0.9 (0.8), [0.1], n=99 | Mean (SE) change from baseline [effect size, Cohen d] 1.1 (−0.8), [0.1], n=98 | Adjusted for baseline score Between group, mean change (95%CI) 0.3 (−1.7 to 2.4), n=197 | between group change (adjusted scores) p=0.742 |  |
| **short term post-surgery (3mths)** | | | | | | |
| Nielsen 2010  n=56 | Quality of life survey 15D (0 - 1 best) | median (range) 0.90 (0.73–1.00), n=28 | median (range) 0.89 (0.62–1.00), n=28 | - | no reported difference |  |
| Rolving 2016b  n=82 | EQ5D (-0.594 worst - 1 best) | **change from baseline mean (95% CI) 0.160 (0.109, 0.210), n = 56*** | **change from baseline mean (95% CI) 0.064 (0.007, 0.135), n = 26*** | **Between group change, mean (95%CI) 0.095 (0.008, 0.193), n = 82*** | **between group difference p=0.034*** |  |
| Lindback 2018  n=197^1^ | EQ5D (-0.594 worst - 1 best) | Mean (SE) change from baseline [effect size, Cohen d] 0.267 (0.0), [1.0], n=99 | Mean (SE) change from baseline [effect size, Cohen d] 0.281 (0.0), [1.0], n=98 | Adjusted for baseline score Between group, mean change (95%CI) 0.004 (-0.1 to 0.1), n=197 | between group change (adjusted scores) p=0.902 |  |
| **medium term post-surgery (6mths)** | | | | | | |
| Nielsen 2010  n=56 | Quality of life survey 15D (0 - 1 best) | median (range) 0.91 (0.73–1.00), n=28 | median (range) 0.90 (0.69–1.00), n=28 | - | no reported difference |  |
| Rolving 2016b  n=83 | EQ5D (-0.594 worst - 1 best) | change from baseline mean (95% CI) 0.143 (0.081, 0.201), n = 58 | change from baseline mean (95% CI) 0.124 (0.035, 0.213), n = 25 | Between group change, mean (95%CI) 0.012 (0.091, 0.130), n = 83 | between group difference p=0.72 |  |
| **long term post-surgery (12mths)** | | | | | | |
| Rolving 2016b  n=84 | EQ5D (-0.594 worst - 1 best) | change from baseline mean (95% CI) 0.135 (0.079. 0.191), n = 56 | change from baseline mean (95% CI) 0.129 (0.062, 0.195), n = 28 | Between group change,  mean (95%CI) 0.006 (0.085, 0.097), n = 84 | between group difference p=0.89 |  |
| Lindback 2018  n=197^1^ | EQ5D (-0.594 worst - 1 best) | Mean (SE) change from baseline [effect size, Cohen d] 0.280 (0.0), [1.0], n=99 | Mean (SE) change from baseline [effect size, Cohen d]  0.329 (0.0), [1.2], n=98 | Adjusted for baseline score Between group, mean change (95%CI) -0.030 (-0.1 to 0.0), n=197 | between group change (adjusted scores) p=0.455 |  |
| Lindback 2018  n=197^1^ | Short Form (SF-36) Physical Component Score (0 - 100 best) | Mean (SE) change from baseline [effect size, Cohen d] 10.0 (1.2), [1.0], n=99 | Mean (SE) change from baseline [effect size, Cohen d] 12.5 (1.3), [1.2], n=98 | Adjusted for baseline score Between group, mean change (95%CI) -1.4 (-4.8 to 1.9), n=197 | between group change (adjusted scores) p=0.404 |  |
| Lindback 2018  n=197^1^ | Short Form (SF-36) Mental Component Score (0 - 100 best) | Mean (SE) change from baseline [effect size, Cohen d] 3.9 (1.1), [0.3], n=99 | Mean (SE) change from baseline[ effect size, Cohen d] 6.6 (1.1), [0.6], n=98 | Adjusted for baseline score Between group, mean change (95%CI) -1.6 (-4.0 to 0.8), n=197 | between group change (adjusted scores) p=0.190 |  |
| **Fear Avoidance** | | | | | | |
| **post-prehab / pre-surgery** | | | | | | |
| Lindback 2018  n=197^1^ | FABQ -  physical activity (0 - 24 worst) | Mean (SE) change from baseline [effect size, Cohen d] -2.5 (0.5), [-0.4], n=99 | Mean (SE) change from baseline [effect size, Cohen d] -0.8 (0.6), [-0.1], n=98 | **Adjusted for baseline score Between group, mean change (95%CI) -1.7 (-3.1 to -0.4), n=197*** | **between group change (adjusted scores) p=0.014*** |  |
| Marchand 2021  n=55 | Tampa Scale of Kinesiophobia (17 best - 68 worst) | mean ± SD (95% CI) 44.2 ± 7.7 (41.3 to 47.1), n=29 | mean ± SD (95% CI) 47.6 ± 8.0 (44.4 to 50.8), n=26 | - | p=0.90 (main effect of group) p=0.02 (group x time interaction, not significant with Bonferroni correction) |  |
| **short term post-surgery (3mths)** | | | | | | |
| Rolving 2015  n=73 | FABQ -  physical activity (0 - 24 worst) | median (IQR) change score -1.0 (-3.0, 1.0), n = 50 | median (IQR) change score 0.0 (5.0, 3.0), n = 23 | - | between group difference p=0.54 |  |
| Marchand 2021  n=47 | Tampa Scale of Kinesiophobia (17 best - 68 worst) | mean ± SD (95% CI) 37.9 ± 7.8 (34.7 to 41.1), n=24 | mean ± SD (95% CI) 41.3 ± 7.3 (38.1 to 44.5), n=23 | - | p=0.48 (main effect of group) p=0.78 (group x time interaction)^2^ |  |
| **medium term post-surgery (6mths)** | | | | | | |
| Rolving 2015  n=73 | FABQ -  physical activity (0 - 24 worst) | **median (IQR) change score -3.0 (-5.0, 1.0), n = 51*** | **median (IQR) change score  0.0 (2.0, 3.0), n = 22*** | **-** | **between group difference p=0.01*** |  |
| Marchand 2021  n=43 | Tampa Scale of Kinesiophobia (17 best - 68 worst) | mean ± SD (95% CI) 37.6 ± 7.6 (34.5 to 40.7) n=24 | mean ± SD (95% CI) 40.4 ± 10.7 (35.5 to 45.3), n=19 | - | p=0.48 (main effect of group) p=0.78 (group x time interaction)^2^ |  |
| **long term post-surgery (12mths)** | | | | | | |
| Rolving 2015  n=75 | FABQ -  physical activity (0 - 24 worst) | median (IQR) change score -3.0 (-7.0, 0.0), n = 51 | median (IQR) change score -2.5 (-6.5, 1.0), n = 24 | - | between group difference p=0.52 |  |
| Lindback 2018  n=197^1^ | FABQ -  physical activity (0 - 24 worst) | Mean (SE) change from baseline [effect size, Cohen d] -5.0 (0.8), [-0.8], n=99 | Mean (SE) change from baseline [effect size, Cohen d] -5.7 (0.8), [-0.9], n=98 | Adjusted for baseline score Between group, mean change (95%CI)  0.7 (-1.2 to 2.6), n=197 | between group change (adjusted scores) p=0.443 |  |
| **Depression / Anxiety** | | | | | | |
| **post-prehab / pre-surgery** | | | | | | |
| Lindback 2018  n=197^1^ | Hospital Anxiety & Depression Scale (HADS) - anxiety scale (0 –21 worst) | Mean (SE) change from baseline [effect size, Cohen d] -0.1 (0.2), [0.0], n=99 | Mean (SE) change from baseline [effect size, Cohen d] -0.1 (0.2), [0.0], n=98 | Adjusted for baseline score Between group, mean change (95%CI) -0.3 (-1.0 to 0.374), n=197 | between group change (adjusted scores) p=0.383 |  |
| Lindback 2018  n=197^1^ | Hospital Anxiety & Depression Scale (HADS)  depression scale (0 –21 worst) | Mean (SE) change from baseline [effect size, Cohen d] 0.0 (0.2), [0.0], n=99 | Mean (SE) change from baseline [effect size, Cohen d] 1.0 (0.2), [0.3], n=98 | **Adjusted for baseline score Between group, mean change (95%CI) -1.0 (-1.7 to -0.4), n=197*** | **between group change (adjusted scores) p=0.002*** |  |
| Marchand 2021  n=55 | Beck Depression inventory (0 - 63 worst) | mean ± SD (95% CI) 4.3 ± 5.3 (2.3 to 6.3), n=29 | mean ± SD (95% CI) 5.3 ± 5.2 (3.2 to 7.3), n=26 | - | p=0.28 (main effect of group) p=0.97 (group x time interaction) |  |
| **short term post-surgery (3mths)** | | | | | | |
| Lindback 2018  n=197^1^ | Hospital Anxiety & Depression Scale (HADS)  anxiety scale (0 –21 worst) | Mean (SE) change from baseline [effect size, Cohen d] -0.9 (0.3), [-0.2], n=99 | Mean (SE) change from baseline [effect size, Cohen d] -1.7 (0.3), [-0.5], n=98 | Adjusted for baseline score Between group, mean change (95%CI) 0.6 (-0.2 to 1.4), n=197 | between group change (adjusted scores) p=0.155 |  |
| Lindback 2018  n=197^1^ | Hospital Anxiety & Depression Scale (HADS)  depression scale (0 –21 worst) | Mean (SE) change from baseline [effect size, Cohen d] -0.9 (0.3), [-0.3], n=99 | Mean (SE) change from baseline [effect size, Cohen d] -1.0 (0.3), [-0.3], n=98 | Adjusted for baseline score Between group, mean change (95%CI) 0.064 (-0.7 to 0.8), n=197 | between group change (adjusted scores) p=0.863 |  |
| Marchand 2021  n=47 | Beck Depression inventory (0 - 63 worst) | mean ± SD (95% CI) 2.7 ± 3.5 (1.3 to 4.2), n=24 | mean ± SD (95% CI) 3.9 ± 4.6 (1.9 to 5.9), n=23 | - | p=0.87 (main effect of group) p=0.87 (group x time interaction)^2^ |  |
| **medium term post-surgery (6mths)** | | | | | | |
| Marchand 2021  n=43 | Beck Depression inventory (0 - 63 worst) | mean ± SD (95% CI) 1.7 ± 2.0 (0.8 to 2.5), n=24 | mean ± SD (95% CI) 3.4 ± 3.3 (1.9 to 4.9), n=19 | - | p=0.87 (main effect of group) p=0.87 (group x time interaction)^2^ |  |
| **long term post-surgery (12mths)** | | | | | | |
| Lindback 2018  n=197^1^ | Hospital Anxiety & Depression Scale (HADS) - anxiety scale (0 –21 worst) | Mean (SE) change from baseline [effect size, Cohen d] -0.1 (0.4), [0.0], n=99 | Mean (SE) change from baseline [effect size, Cohen d] -1.0 (0.4), [-0.2], n=98 | Adjusted for baseline score Between group, mean change (95%CI) 0.7 (-0.3 to 1.7), n=197 | between group change (adjusted scores) p=0.143 |  |
| Lindback 2018  n=197^1^ | Hospital Anxiety & Depression Scale (HADS) - depression scale (0 –21 worst) | Mean (SE) change from baseline [effect size, Cohen d] -0.5 (0.4), [-0.1], n=99 | Mean (SE) change from baseline [effect size, Cohen d] -0.6 (0.4), [-0.2], n=98 | Adjusted for baseline score Between group, mean change (95%CI) 0.1 (-0.8 to 1.0), n=197 | between group change (adjusted scores) p=0.809 |  |
| **Other (psychological)** | | | | | | |
| **post-prehab / pre-surgery** | | | | | | |
| Lindback 2018  n=197^1^ | Self-Efficacy Scale (0 – 200 best) | Mean (SE) change from baseline [effect size, Cohen d] 1.7 (2.4), [0.0], n=99 | Mean (SE) change from baseline [effect size, Cohen d] -5.7 (2.4), [-0.2], n=98 | **Adjusted for baseline score Between group, mean change (95%CI) 8.7 (2.2 to 15.2), n=197*** | **between group change (adjusted scores) p=0.009*** |  |
| **short term post-surgery (3mths)** | | | | | | |
| Lindback 2018  n=197^1^ | Self-Efficacy Scale (0 – 200 best) | Mean (SE) change from baseline [effect size, Cohen d] 14.8 (3.5), [0.4], n=99 | Mean (SE) change from baseline [effect size, Cohen d] 24.3 (3.6), [0.7], n=98 | Adjusted for baseline score Between group, mean change (95%CI) -6.0 (-15.1 to 3.2), n=197 | between group change (adjusted scores) p=0.200 |  |
| Rolving 2015  n=77 | Coping Strategies Questionnaire  catastrophizing subscale (0 - 36 worst) | median (IQR) change score  -5.0 (-10, 2.0), n = 51 | median (IQR) change score -2.0 (-8.0, 0.0), n = 26 | - | between group difference p=0.47 |  |
| **medium term post-surgery (6mths)** | | | | | | |
| Rolving 2015  n=79 | Coping Strategies Questionnaire  catastrophizing subscale (0 - 36 worst) | **median (IQR) change score -7.5 (-12, 0.0), n = 54*** | **median (IQR) change score -2.0 (-5.0, 3.0), n = 25*** | **-** | **between group difference p=0.04*** |  |
| **long term post-surgery (12mths)** | | | | | | |
| Rolving 2015  n=75 | Coping Strategies Questionnaire  catastrophizing subscale (0 - 36 worst) | median (IQR) change score -5.0 (-10.0, 0.0), n = 49 | median (IQR) change score  -5.5 (-11.0, -1.0), n = 26 | - | between group difference p=0.51 |  |
| Lindback 2018  n=197^1^ | Self-Efficacy Scale (0 – 200 best) | Mean (SE) change from baseline [effect size, Cohen d] 19.1 (3.4), [0.5], n=99 | Mean (SE) change from baseline [effect size, Cohen d] 28.4 (3.4), [0.8], n=98 | Adjusted for baseline score Between group, mean change (95%CI) -6.9 (-16.2 to 2.3), n=197 | between group change (adjusted scores) p=0.140 |  |
| **Physical activity (self-reported)** | | | | | | |
| **post-prehab / pre-surgery** | | | | | | |
| Lindback  2018  n=197^1^ | count (responses to a question with five answer  options) | not reported | not reported | **between group difference Cramer V effect size 0.391*** | **between group difference p<0.001*** |  |
| **long term post-surgery (12mths)** | | | | | | |
| Lindback 2018  n=197^1^ | count (responses to a question with five answer options) | not reported | not reported | **Cramer V effect size 0.26*** | **between group difference p=0.020*** |  |
| **Walking distance (self-reported)** | | | | | | |
| **post-prehab / pre-surgery** | | | | | | |
| Fors 2019 (Lindback  study)  n=142 | item four Oswestry  Disability Index (ODI)- how pain affects walking distance | **n=67, n(%) doesn't prevent walking:**  **baseline 16 (24%); FU 13 (20%)  prevents walking >1km:**  **baseline 19 (28%); FU 32 (47%)  prevents walking >500m:**  **baseline 16 (24%); FU 13 (20%)  prevents walking >100m:**  **baseline 15 (22%); FU 9 (13%)  walk using a stick/crutches:**  **baseline 1 (2%); FU 0 in bed most of time:**  **baseline 0; FU 0** | **n=75, n(%) doesn't prevent walking:**  **baseline 10 (13%); FU 8 (10%) prevents walking >1km:**  **baseline 28 (38%); FU 27 (36%) prevents walking >500m:**  **baseline 23 (31%); FU 21 (28%) prevents walking >100m:**  **baseline 10 (13%); FU 11 (15%) walk using a stick/crutches;**  **baseline 4 (5%); FU 8 (11%)  in bed most of time:**  **baseline 0; FU 0** | - | **between group change p=0.007*** |  |
| Fors 2019 (Lindback  study)  n=148 | question from SWESPINE (Swedish spine regsister) “How far can you walk at normal walk speed?” | **n (%), n=71 <100 m:**  **baseline 2 (3%); FU 4 (5%) 100-500 m:**  **baseline 34 (48%); FU 25 (35%) 0.5 km – 1 km:**  **baseline 12 (17%); FU 11 (16%) >1 km:**  **baseline 23 (32%); FU 31 (44%)** | **n (%), n=77 <100 m:**  **baseline 6 (8%); FU 7 (9%) 100-500 m:**  **baseline 31 (40%); FU 32 (41%) 0.5 km – 1 km:**  **baseline 22 (29%); FU 22 (29%) >1 km:**  **baseline 18 (23%); FU 16 (21%)** | - | **between group change p=0.028*** |  |
| *analysis statistically significant (p<0.05)  1 used imputed data to include all randomised subjects; n(follow-ups completed) is baseline: 99 intervention & 98 control; post-prehab/pre-surgery: 80 intervention & 89 control (169 total) patients (14% dropout); 3 mths: 72 intervention & 82 control (154 total) patients (22% dropout); 12 mths: 62 intervention & 78 control (140 total) patients (29% dropout)  2 for post-op time points, time & group x time analyses compared 3 & 6 mth scores to 6 wk post-surgery scores, not baseline  3 main results paper for Rolving (2015) reported median (IQR) change from baseline whereas economic evaluation paper (2016b) reported mean (95% CI) change from baseline  4 results statistically significant in Rolving 2016b but not 2015 paper  5 discrepancy between text in statistical methods section and figures provided in graph (figure 2) | | | | | | |

G. Summary of study results - objective outcomes

|  | **measurement tool** | **Intervention** | **Control** | **between group change** | **p-value** |
| --- | --- | --- | --- | --- | --- |
| **Walking** | | | | | |
| **post-prehab / pre-surgery** | | | | | |
| Fors 2019 (Lindback study)  n=149 | 10 metre walk test, speed (m/s) | Mean (SD) change from baseline [effect size, Cohen d], m/s normal speed: -0.09 (0.14) [- 0.34], n=72 fastest speed: - 0.12 (0.22) [- 0.29], n=72 | Mean (SD) change from baseline [effect size, Cohen d], m/s normal speed: - 0.01 (0.17) [- 0.04], n=77 fastest speed: 0.05 (0.36) [0.12], n=75 | **Between group change Mean difference (95% CI), m/s normal speed: 0.07 (0.02 to 0.12)* fastest speed: 0.21 (0.07 to 0.26)*** | **Between group change normal speed: p=0.005* fastest speed: p<0.001*** |
| Marchand 2021  n=54 | time to first symptoms (seconds) | **mean ± SD (95% CI) 158.8 ± 102.4 (119.8 to 197.7), n=29*** | **mean ± SD (95% CI) 79.4 ± 87.4 (43.3 to 115.4), n=25*** | **-** | **p=0.01* (main effect of group) p=0.08 (group x time interaction)** |
| Marchand 2021  n=54 | total ambulation time (seconds) | **mean ± SD (95% CI) 231.7 ± 92.2 (195.9 to 267.5), n=29*** | **mean ± SD (95% CI) 146.3 ± 104.7 (103.1 to 189.5), n=25*** | **-** | **p=0.03* (main effect of group) p=0.02 (group x time interaction)** |
| **short term post-surgery (6 weeks)** | | | | | |
| Marchand 2021  n=51 | time to first symptoms (seconds) | mean ± SD (95% CI) 223.1 ± 106.5 (182.6 to 263.6), n=30 | mean ± SD (95% CI) 199.6 ± 122.8 (143.7 to 255.5), n=21 | - | no reported difference |
| Marchand 2021  n=51 | total ambulation time (seconds) | mean ± SD (95% CI) 262.2 ± 78.5 (232.3 to 292.0), n=30 | mean ± SD (95% CI) 262.0 ± 78.1 (226.5 to 297.6), n=21 | - | no reported difference |
| **Function** | | | | | |
| **post-prehab / pre-surgery** | | | | | |
| Nielsen 2010  n=60 | sit-to-stand time to do 5 reps (seconds) | median (range) 14 (8–22), n=28 | median (range) 18 (8–31), n=32 | **-** | no reported difference |
| Nielsen 2010  n=60 | Timed  up-and-go 1 rep (seconds) | median (range) 9 (6–31), n=28 | median (range) 11 (7–27), n=32 | **-** | no reported difference |
| Marchand 2021  n=17 | sit-to-stand count (reps in 30secs) | mean ± SD (95% CI) 12.1 ± 3.6 (9.4 to 14.9), n=9 | mean ± SD (95% CI) 8.4 ± 2.2 (6.8 to 10.1), n=8 | **-** | p=0.14 (main effect of group) p=0.01 (group x time interaction) |
| Marchand 2021  n=17 | Timed  up-and-go 1 rep (seconds) | mean ± SD (95% CI) 7.5 ± 1.9 (6.0 to 8.9), n=9 | mean ± SD (95% CI) 9.7 ± 2.6 (7.7 to 11.7), n=8 | - | p=0.15 (main effect of group) p=0.12 (group x time interaction) |
| **short term post-surgery (4-6 weeks)** | | | | | |
| Nielsen 2010  n=56 | sit-to-stand 5 reps (seconds) | median (range) 12 (8–28), n=28 | median (range) 14 (5–32), n=28 | **-** | no reported difference |
| Nielsen 2010  n=56 | Timed  up-and-go 1 rep (seconds) | median (range)  8 (5–31), n=28 | median (range) 9 (5–18), n=28 | **-** | no reported difference |
| Marchand 2021  n=19 | sit-to-stand count (reps in 30secs) | mean ± SD (95% CI) 11.8 ± 2.2 (10.4 to 13.1), n=13 | mean ± SD (95% CI) 12.0 ± 2.1 (9.8 to 14.2), n=6 | **-** | no reported difference |
| Marchand 2021  n=19 | Timed  up-and-go 1 rep (seconds) | mean ± SD (95% CI) 6.7 ± 1.3 (5.9 to 7.5), n=13 | mean ± SD (95% CI) 8.0 ± 2.7 (5.2 to 10.9), n=6 | - | no reported difference |
| **short term post-surgery (3mths)** | | | | | |
| Nielsen 2010  n=56 | sit-to-stand time to do 5 reps (seconds) | median (range) 11 (8–23), n=28 | median (range) 12 (8–33), n=28 | **-** | no reported difference |
| Nielsen 2010  n=56 | Timed up-and-go time to do 1 rep (seconds) | median (range) 8 (5–17), n=28 | median (range) 9 (5–14), n=28 | **-** | no reported difference |
| **medium term post-surgery (6mths)** | | | | | |
| Nielsen 2010  n=56 | sit-to-stand time to do 5 reps (seconds) | median (range) 12 (7–20), n=28 | median (range) 13 (7–27), n=28 | **-** | no reported difference |
| Nielsen 2010  n=56 | Timed up-and-go time to do 1 rep (seconds) | median (range) 8 (5–18), n=28 | median (range) 8 (5–13), n=28 | **-** | no reported difference |
| *analysis statistically significant (p<0.05)  1 used imputed data to include all randomised subjects; n (followups completed) is baseline: 99 intervention & 98 control; post-prehab/pre-surgery: 80 intervention & 89 control (169 total) patients (14% dropout); 3 mths: 72 intervention & 82 control (154 total) patients (22% dropout); 12 mths: 62 intervention & 78 control (140 total) patients (29% dropout)  2 for post-op time points, time & group x time analyses compared 3 & 6 mth scores to 6 wk post-surgery scores, not baseline  3 main results paper for Rolving (2015) reported median (IQR) change from baseline whereas economic evaluation paper (2016b) reported mean (95% CI) change from baseline  4 results statistically significant in Rolving 2016b but not 2015 paper  5 discrepancy between text in statistical methods section and figures provided in graph (figure 2) | | | | | |

H. Summary of study results – perioperative outcomes

|  | **measurement tool** | **Intervention** | **Control** | **between group change** | **p-value** | **comments** |
| --- | --- | --- | --- | --- | --- | --- |
| **Hospital Stay** | |  |  |  |  |  |
| Nielsen 2010 n=60 | hospital stay (days) | **median (range) 5 days (3–9), n=28*** | **median (range) 7 days (5-15), n=32*** | **-** | **between group difference p=0.007*** |  |
| Rolving 2016a n=90 | hospital stay (days) | median (IQR) [range] 5 days (4, 6),[ 3-9], n=59 | median (IQR) [range] 4 days (4, 6), [3-10], n=31 | - | between group difference p=0.46 |  |
| Marchand 2021 n=64 | hospital stay (days) | mean ± SD 4.1 ± 3.2, n=34 | mean ± SD 4.5 ± 2.0, n=30 | - | between group difference p=0.58 |  |
| **Adverse events** | |  |  |  |  |  |
| Nielsen 2010 n=60 | count, n (%) | 3/28 (11%) | 1/32 (3%) | - | not reported |  |
| **Analgesia** |  |  |  |  |  |  |
| Rolving 2016a n=90 | count (extra 'rescue' analgesic use [daily morphine-equivalent doses] first 5 days post-surgery) | median (IQR) 142.5 (70, 275), n = 59 | median (IQR) 196.8 (145, 345), n = 31 | - | between group difference p=0.23 |  |
| Rolving 2016b n=90 | count (prescription medicine [packages first year post-op) | mean (95% CI) 28.0 (21.4, 34.6), n = 59 | mean (95% CI) 21.6 (13.4, 29.9), n = 31 | between group mean (95% CI) difference 6.4 (4.1; 16.9), n=90 | between group difference p=0.23 |  |
| **Complications** | |  |  |  |  |  |
| Nielsen 2010 n=60 | count (number of patients with complications [major+minor]) | 12/28 (43%) | 12/32 (38%) | - | no reported difference |  |
| Nielsen 2010 n=60 | count (days extra stay due to complications) | 18 days, n=28 | 30 days, n=32 | - | between group difference p=0.06 or p<0.01? |  |
| **Return to Work** | |  |  |  |  |  |
| Nielsen 2008 n=60 | count (days post-surgery) | mean/median? 77 days (14–90), n=28 | mean/median? 88 days (54–90), n=32 | - | between grouo difference p = 0.092 |  |
| Rolving 2015 n=69 | count (number of patients in workforce 12mths/baseline) | n (%) 22/43 (51%) | n (%) 16/26 (62%) | - | no reported difference |  |
| Rolving 2015 n=69 | count (patients returned to work at 12mths/ in- workforce baseline) | n (%) 18/43 (42 %) | n (%) 11/26 (42 %) | - | no reported difference |  |
| Rolving 2015 n=69 | count (weeks sick leave during first year after surgery) | median (IQR) 31 (16, 52), n=43 | median (IQR) 39 (9, 52), n=26 | - | between group difference p = 0.87 |  |
| **Healthcare Resource Use** | |  |  |  |  |  |
| Rolving 2016b n=90 | number of visits during 12 mths after surgery | mean number of visits (95% CI), n=59 *GP:* 16.5 (13.6, 19.5) *Specialist:* 1.3 (0.6, 2.0) *Physio:* 6.2 (1.9; 10.6) *bed days:* 7.7 (5.9, 9.5) *outpatients:* 7.8 (6.1, 9.6) *A&E:* 0.12 (0.04, 0.2) | mean number of visits (95% CI), n=31 *GP:* 17.4 (14.1, 20.7) *Specialist:* 1.3 (0.3, 2.2) *Physio:* 6.2 (0.9, 11.5) *bed days:* 6.8 (4.8, 8.9) *outpatients:* 7.5 (4.9, 10.2) *A&E:* 0.16 (0.03, 0.3) | between group mean difference (95% CI), n=90 *GP:* -0.9 (-0.3; 3.6) *Specialist:* 0.0 (-1.1; 1.1) *Physio:* 0.0 (-7.5; 7.5) *bed days:* 0.9 (-1.7; 3.5) *outpatients:* 0.3 (-2.6; 3.4) *A&E:* -0.04 (-0.2; 0.1) | between group difference *GP:* p=0.7 *Specialist:* p=0.99 *Physio:* p=0.99 *bed days:* p=0.66 *outpatients:* p=0.21 *A&E:* p=0.59 |  |
| **Inpatient Mobility** | |  |  |  |  |  |
| Nielsen 2010 n=60 | Stay until reaching all mobility milestones (days) | **median (range) 4 days- (1-6), n=28*** | **median (range) 6 days- (3-13)*, n=32*** | **-** | **bewteen group difference p=0.001*** |  |
| Rolving 2016a n=90 | Cumulated Ambulation Score - count(patients able to perform without support on day 3 postop) | **n (%) *walking:* 43/59 (73%)* *sit-to-stand:* 58/59 (98%)* *in & out of bed:* 58/59 (98%)*** | **n (%) *walking:* 15/31 (48%)* *sit-to-stand:* 26/31 (84%)* *in & out of bed:* 26/31 (84%)*** | **-** | **between group difference *walking:* p=0.02* *sit-to-stand:* p=0.017* *in & out of bed:* p=0.017*** |  |
| **LBP 1st week post-surgery** | |  |  |  |  |  |
| Rolving 2016a n=65 | daily back pain days 1st week - 11 point NRS (0 no pain - 10 worst) | median (IQR) 5.4 (4.0, 6.5), n = 44 | median (IQR) 5.3 (4.0, 6.1), n = 21 | - | between group difference p=0.74 |  |
| **Change in condition pre-surgery / post-prehabilitation** | | | |  |  |  |
| Lindback 2018 n=169^5^ | Patient Global Impression of Change | ***Improved:* 49%* *No change:* 38% *Worse:* 13%*** | ***Improved:* 17%* *No change:* 41% *Worse:* 42%*** | **-** | **between group difference p<0.001*** | only % provided; reported no between group difference at 3 & 12mths but no data provided |
| Marchand 2021 n=? | Clinical Global Impression - Improvement scale | mean ± SD 2.9 ± 1.3 | mean ± SD  4.5 ± 1.0 | - | not reported | details re: scale & n(participants) not reported |
| Marchand 2021 n=? | patient perception of treatment effect 7-point scale (proportion improved/worsened) | ***improved:* 69%* *worsened:* 13%*** | ***improved:* 11.5%* *worsened:* 46%*** | **-** | **between group difference p<0.001*** | only % provided |
| **Satisfaction post-surgery** | |  |  |  |  |  |
| Nielsen 2010 n=60 | satisfaction with overall treatment & outcome - 5-point scale (1mth post-surgery) | ***very satisfed:* 15/28 (54%)*** | ***vey satisfied:* 7/32 (22%)*** | **-** | **between group difference p=0.02*** | data for other categories not reported |
| Marchand 2021 n=? | Satisfaction with surgical results (back/leg pain - 0-100% scale) | mean ± SD *post-op back pain:* 84.4% ± 22.2 *post-op leg pain:* 82.3% ± 23.4 | mean ± SD *post-op back pain:* 84.0% ± 22.5 *post-op leg pain:* 84.6% ± 23.9 | - | between group difference p=0.23 (LBP) p=0.34 (leg pain) |  |
| *analysis statistically significant (p<0.05)  1 used imputed data to include all randomised subjects; n(follow-ups completed) is baseline: 99 intervention & 98 control; post-prehab/pre-surgery: 80 intervention & 89 control (169 total) patients (14% dropout); 3 mths: 72 intervention & 82 control (154 total) patients (22% dropout); 12 mths: 62 intervention & 78 control (140 total) patients (29% dropout)  2 for post-op time points, time & group x time analyses compared 3 & 6 mth scores to 6 wk post-surgery scores, not baseline  3 main results paper for Rolving (2015) reported median (IQR) change from baseline whereas economic evaluation paper (2016b) reported mean (95% CI) change from baseline  4 results statistically significant in Rolving 2016b but not 2015 paper  5 discrepancy between text in statistical methods section and figures provided in graph (figure 2) | | | | | | |

I. Summary of components featured in successful interventions – disability (post-prehabilitation / pre-surgery)

| component | Nielsen (n=73) | Rolving^1^ (n=96) | Lindback (n=197) | Marchand (n=68) | frequency component featured in successful intervention / total number of studies with successful interventions | frequency component featured in UNsuccessful interventions / total number of studies with UNsuccessful interventions | |
| --- | --- | --- | --- | --- | --- | --- | --- |
| CBT / other behavioural | **✓** | NA | **✓** |  | 2/2 | 0/1 | |
| Exercise | **✓** |  | **✓** | **ND** | 2/2 | 1/1 | |
| CV | **✓** |  | **✓** |  | 2/2 | 0/1 | |
| strength | **✓** |  | **✓** | **ND** | 2/2 | 1/1 | |
| trunk / core | **✓** |  | NR | **ND** | 1/2 | 1/1 | |
| lower limb |  |  | NR | **ND** | 0/2 | 1/1 | |
| Intensity/ effort considered | NR |  | **✓** | **ND** | 1/2 | 1/1 | |
| Exercise progression | NR |  | **✓** | **ND** | 1/2 | 1/1 | |
| structured programme | **✓** |  | **✓** | **ND** | 2/2 | 1/1 | |
| supervised |  |  | **✓** | **ND** | 1/2 | 1/1 | |
| unsupervised | **✓** |  | **✓** (HEP) |  | 2/2 | 0/1 | |
| individual | **✓** |  | **✓** | **ND** | 2/2 | 1/1 | |
| tailored / individualised | **✓** |  | **✓** | **ND** | 2/2 | 1/1 | |
| home | **✓** |  | **✓** (HEP) |  | 2/2 | 0/1 | |
| gym / clinic / community |  |  | **✓** | **ND** | 1/2 | 1/1 | |
| 6-8 weeks | **✓** |  |  | **ND** | 1/2 | 1/1 | |
| x 3-4 / week |  |  |  | **ND** | 0/2 | 1/1 | |
| daily | **✓** |  | **✓** (HEP) |  | 2/2 | 0/1 | |
| ≤30 minutes | **✓** |  |  | **ND** | 1/2 | 1/1 | |
| Adherence / compliance strategies | **✓** |  | **✓** | NR | 2/2 | 0/1 | |
| Physiotherapist | **✓** |  | **✓** |  | 2/2 | 0/1 | |
| Kinesiologists |  |  |  | **ND** | 0/2 | 1/1 | |
| evidence other populations | NR |  | **✓**(TBC)^2^ | **ND** | 1/2 | 1/1 | |
| info re: surgery etc | **✓** |  | **✓** | **ND** | 2/2 | 1/1 | |
| **✓ =** featured in successful intervention; ND = featured in unsuccessful interventions; NA = not applicable; NR = not reported HEP = home exercise programme. ^1^Rolving did not record post-prehab/pre-surgery outcomes; ^2^TBC - Treatment-based classification - 1 of either traction, pelvic floor/trunk motor control exercises or specific symptom reducing directional movements | | | | | | |  |

J. Summary of components featured in successful interventions – disability (3 months)

| component | Nielsen (n=73) | Rolving (n=96) | Lindback (n=197) | Marchand (n=68) | frequency component featured in successful interventions / n(studies with successful interventions) | frequency component featured in UNsuccessful interventions / n(studies with UNsuccessful interventions) |  |
| --- | --- | --- | --- | --- | --- | --- | --- |
| CBT / other behavioural | **ND** | **✓** | **ND** |  | 1/1 | 2/3 |  |
| Exercise | **ND** | NA | **ND** | **ND** | 0/1 | 3/3 |  |
| CV | **ND** | NA | **ND** |  | 0/0 | 2/3 |  |
| strength | **ND** | NA | **ND** | **ND** | 0/0 | 3/3 |  |
| trunk / core | **ND** | NA | NR | **ND** | 0/0 | 2/3 |  |
| Intensity/ effort considered | NR | NA | **ND** | **ND** | 0/0 | 2/3 |  |
| Exercise progression | NR | NA | **ND** | **ND** | 0/0 | 2/3 |  |
| structured programme | **ND** | **✓** | **ND** | **ND** | 1/1 | 3/3 |  |
| unstructured programme |  |  | **ND** (HEP) |  | 0/1 | 2/3 |  |
| supervised |  | **✓** | **ND** | **ND** | 1/1 | 2/3 |  |
| unsupervised | **ND** |  | **ND** (HEP) |  | 0/1 | 2/3 |  |
| group |  | **✓** |  |  | 1/1 | 0/3 |  |
| individual | **ND** |  | **ND** | **ND** | 0/1 | 3/3 |  |
| tailored / individualised | **ND** |  | **ND** | **ND** | 0/1 | 3/3 |  |
| standardised |  | **✓** |  |  | 1/1 | 0/3 |  |
| home | **ND** |  | **ND** (HEP) |  | 0/1 | 2/3 |  |
| gym / clinic / community |  | **✓** | **ND** | **ND** | 1/1 | 2/3 |  |
| 3-5 weeks |  | **✓** |  |  | 1/1 | 0/3 |  |
| 6-8 weeks | **ND** |  |  | **ND** | 0/1 | 2/3 |  |
| daily | **ND** |  | **ND** (HEP) |  | 0/1 | 2/3 |  |
| ≤30 minutes | **ND** |  |  | **ND** | 0/1 | 2/3 |  |
| ≥60 minutes |  | **✓** |  |  | 1/1 | 0/3 |  |
| Adherence / compliance strategies | **ND** |  | **ND** | NR | 0/1 | 2/3 |  |
| Physiotherapist | **ND** | **✓** | **ND** |  | 1/1 | 2/3 |  |
| Psychologist |  | **✓** |  |  | 1/1 | 0/3 |  |
| medic / surgeon |  | **✓** |  |  | 1/1 | 0/3 |  |
| Occupational therapist |  | **✓** |  |  | 1/1 | 0/3 |  |
| Social worker |  | **✓** |  |  | 1/1 | 0/3 |  |
| former patient |  | **✓** |  |  | 1/1 | 0/3 |  |
| intervention delivery training | NR | **✓** | **ND** | NR | 1/1 | 1/3 |  |
| evidence other populations | NR | **✓** | **ND** (TBC)^2^ | **ND** | 1/1 | 2/3 |  |
| info re: surgery etc | **ND** | **✓** | **ND** | **ND** | 1/1 | 3/3 |  |
| additional post-op elements^3^ | **ND** | **✓** |  |  | 1/1 | 1/3 |  |
| ✓ = featured in successful intervention; ND = featured in unsuccessful interventions; NA = not applicable; NR = not reported HEP = home exercise programme  ^2^TBC - Treatment-based classification - 1 of either traction, pelvic floor/trunk motor control exercises or specific symptom reducing directional movements; ^3^ Rolving - 3hr CBT refresher session at 3 and 6mths; Nielsen - double physio inpatient rehab time, optimised pain regime, protein supplements, smoking cessation follow-up | | | | | | | |

.

K. Summary of components featured in successful interventions - disability (6 months)

| component | Nielsen (n=73) | Rolving (n=96) | Lindback^1^ (n=197) | Marchand (n=68) | frequency component featured in successful intervention / n(studies with successful interventions) | frequency component featured in UNsuccessful interventions / n(studies with UNsuccessful interventions |
| --- | --- | --- | --- | --- | --- | --- |
| CBT / other behavioural | **ND** | **✓** |  |  | 1/1 | 1/2 |
| Exercise | **ND** |  |  | **ND** | 0/1 | 2/2 |
| strength | **ND** | NA |  | **ND** | 0/0 | 2/2 |
| trunk / core | **ND** | NA |  | **ND** | 0/0 | 2/2 |
| structured programme | **ND** | **✓** |  | **ND** | 1/1 | 2/2 |
| supervised |  | **✓** |  | **ND** | 1/1 | 1/2 |
| group |  | **✓** |  |  | 1/1 | 0/2 |
| individual | **ND** |  |  | **ND** | 0/1 | 2/2 |
| tailored / individualised | **ND** |  |  | **ND** | 0/1 | 2/2 |
| standardised |  | **✓** |  |  | 1/1 | 0/2 |
| gym / clinic / community |  | **✓** |  | **ND** | 1/1 | 1/2 |
| 3-5 weeks |  | **✓** |  |  | 1/1 | 0/2 |
| 6-8 weeks | **ND** |  |  | **ND** | 0/1 | 2/2 |
| ≤30 minutes | **ND** |  |  | **ND** | 0/1 | 2/2 |
| ≥60 minutes |  | **✓** |  |  | 1/1 | 0/2 |
| Physiotherapist | **ND** | **✓** |  |  | 1/1 | 1/2 |
| Psychologist |  | **✓** |  |  | 1/1 | 0/2 |
| medic / surgeon |  | **✓** |  |  | 1/1 | 0/2 |
| Occupational therapist |  | **✓** |  |  | 1/1 | 0/2 |
| Social worker |  | **✓** |  |  | 1/1 | 0/2 |
| former patient |  | **✓** |  |  | 1/1 | 0/2 |
| intervention delivery training | NR | **✓** |  | NR | 1/1 | 0/2 |
| evidence other populations | NR | **✓** |  | **ND** | 1/1 | 1/2 |
| info re: surgery etc | **ND** | **✓** |  | **ND** | 1/1 | 2/2 |
| additional post-op elements | **ND** | **✓** |  |  | 1/1 | 1/2 |
| **✓ =** featured in successful intervention; ND = featured in unsuccessful interventions; NA = not applicable; NR = not reported HEP = home exercise programme  ^1^Marchand did not record 3 month outcomes; ^2^TBC - Treatment-based classification - 1 of either traction, pelvic floor/trunk motor control exercises or specific symptom reducing directional movements; ^3^Rolving - 3hr CBT refresher session at 3 and 6mths; Nielsen - double physio inpatient rehab time, optimised pain regime, protein supplements, smoking cessation follow-up | | | | | | |

L. Summary of components featured in successful interventions - HRQoL (3 months)

| component | Nielsen (n=73) | Rolving (n=96) | Lindback (n=197) | Marchand^1^ (n=68) | frequency component featured in successful intervention / n(studies with successful interventions) | frequency component featured in UNsuccessful interventions / n(studies with UNsuccessful interventions) |
| --- | --- | --- | --- | --- | --- | --- |
| CBT / other behavioural | **ND** | **✓** | **ND** |  | 1/1 | 2/2 |
| Exercise | **ND** |  | **ND** |  | 0/1 | 2/2 |
| CV | **ND** | NA | **ND** |  | 0/0 | 2/2 |
| strength | **ND** | NA | **ND** |  | 0/0 | 2/2 |
| structured programme | **ND** | **✓** | **ND** |  | 1/1 | 2/2 |
| supervised |  | **✓** | **ND** |  | 1/1 | 1/2 |
| unsupervised | **ND** |  | **ND** (HEP) |  | 0/1 | 2/2 |
| group |  | **✓** |  |  | 1/1 | 0/2 |
| individual | **ND** |  | **ND** |  | 0/1 | 2/2 |
| tailored / individualised | **ND** |  | **ND** |  | 0/1 | 2/2 |
| standardised |  | **✓** |  |  | 1/1 | 0/2 |
| home | **ND** |  | **ND** (HEP) |  | 0/1 | 2/2 |
| gym / clinic / community |  | **✓** | **ND** |  | 1/1 | 1/2 |
| 3-5 weeks |  | **✓** |  |  | 1/1 | 0/2 |
| daily | **ND** |  | **ND** (HEP) |  | 0/1 | 2/2 |
| ≥60 minutes |  | **✓** |  |  | 1/1 | 0/2 |
| Adherence / compliance strategies | **ND** |  | **ND** |  | 0/1 | 2/2 |
| Physiotherapist | **ND** | **✓** | **ND** |  | 1/1 | 2/2 |
| Psychologist |  | **✓** |  |  | 1/1 | 0/2 |
| medic / surgeon |  | **✓** |  |  | 1/1 | 0/2 |
| Occupational therapist |  | **✓** |  |  | 1/1 | 0/2 |
| Social worker |  | **✓** |  |  | 1/1 | 0/2 |
| former patient |  | **✓** |  |  | 1/1 | 0/2 |
| intervention delivery training | NR | **✓** | **ND** |  | 1/1 | 1/2 |
| evidence other populations | NR | **✓** | **ND** (TBC)^2^ |  | 1/1 | 1/2 |
| info re: surgery etc | **ND** | **✓** | **ND** |  | 1/1 | 2/2 |
| additional post-op elements^3^ | **ND** | **✓** |  |  | 1/1 | 1/2 |
| **✓ =** featured in successful intervention; ND = featured in unsuccessful interventions; NA = not applicable; NR = not reported HEP = home exercise programme  ^1^Marchand did not record 3 month outcomes; ^2^TBC - Treatment-based classification - 1 of either traction, pelvic floor/trunk motor control exercises or specific symptom reducing directional movements; ^3^Rolving - 3hr CBT refresher session at 3 and 6mths; Nielsen - double physio inpatient rehab time, optimised pain regime, protein supplements, smoking cessation follow-up | | | | | | |

M. Summary of components featured in successful interventions – Hospital stay

| component | Nielsen (n=73) | Rolving (n=96) | Lindback (n=197)^1^ | Marchand (n=68) | frequency component featured in successful intervention / n(studies with successful interventions) | frequency component featured in UNsuccessful interventions / n(studies with UNsuccessful interventions) |
| --- | --- | --- | --- | --- | --- | --- |
| CBT / other behavioural | **✓** | **ND** |  |  | 1/1 | 1/2 |
| Exercise | **✓** |  |  | **ND** | 1/1 | 1/2 |
| CV | **✓** | NA |  |  | 1/1 | 0/1 |
| strength | **✓** | NA |  | **ND** | 1/1 | 1/1 |
| trunk / core | **✓** | NA |  | **ND** | 1/1 | 1/1 |
| lower limb |  | NA |  | **ND** | 0/1 | 1/1 |
| Intensity/ effort considered | NR | NA |  | **ND** | 0/1 | 1/1 |
| Exercise progression | NR | NA |  | **ND** | 0/1 | 1/1 |
| structured programme | **✓** | **ND** |  | **ND** | 1/1 | 2/2 |
| supervised |  | **ND** |  | **ND** | 0/1 | 2/2 |
| unsupervised | **✓** |  |  |  | 1/1 | 0/2 |
| individual | **✓** |  |  | **ND** | 1/1 | 1/2 |
| tailored / individualised | **✓** |  |  | **ND** | 1/1 | 1/2 |
| home | **✓** |  |  |  | 1/1 | 0/2 |
| gym / clinic / community |  | **ND** |  | **ND** | 0/1 | 2/2 |
| 6-8 weeks | **✓** |  |  | **ND** | 1/1 | 1/2 |
| daily | **✓** |  |  |  | 1/1 | 0/2 |
| ≤30 minutes | **✓** |  |  | **ND** | 1/1 | 1/2 |
| 31-60 minutes |  |  |  |  | 0/1 | 0/2 |
| Adherence / compliance strategies | **✓** |  |  | NR | 1/1 | 0/2 |
| Physiotherapist | **✓** | **ND** |  |  | 1/1 | 1/2 |
| Kinesiologists |  | NA |  | **ND** | 0/1 | 1/1 |
| evidence other populations | NR | **ND** |  | **ND** | 0/1 | 2/2 |
| info re: surgery etc | **✓** | **ND** |  | **ND** | 1/1 | 2/2 |
| protein supplements | **✓** |  |  |  | 1/1 | 0/2 |
| optimised pain relief | **✓** |  |  |  | 1/1 | 0/2 |
| additional post-op elements | **✓** | **ND** |  |  | 1/1 | 1/2 |
| **✓ =** featured in successful intervention; ND = featured in unsuccessful interventions; NA = not applicable; NR = not reported; HEP = home exercise programme  ^1^Lindback did not record hospital stay; ^3^Rolving - 3hr CBT refresher session at 3 and 6mths; Nielsen - double physio inpatient rehab time, optimised pain regime, protein supplements, smoking cessation follow-up | | | | | | |
